# Supplementary material for: Mobility can promote the evolution of cooperation via emergent self-assortment dynamics
Source: PLoS Comput Biol. 2017 Sep 8;13(9):e1005732. doi: 10.1371/journal.pcbi.1005732 (PMC5607214; doi:10.1371/journal.pcbi.1005732)
Supplement: S1 Appendix — (PDF) [file pcbi.1005732.s001.pdf]

S1 Appendix Methods

S1.1 System description

Our model framework is based on the individual-based spatially-explicit evolutionary framework developed in [1] and [2]. We simulate a population consisting of N individuals indexed by $i = \{1, 2, ...N\}$. Individuals move in a two-dimensional continuous space of size $L \times L$ with periodic boundary conditions. Periodic boundary means that when an individual crosses any boundary, it re-emerges from the opposite boundary. This can be viewed as a warping of space into the shape of a torus. The distance between two individuals is the shortest distance on the surface of this torus. Such a boundary condition enables us to model a small snapshot of a large system while preserving the essential ecological features of the large system.

The important symbols and values of fixed parameters are summarized in table A.

S1.2 Evolvable traits: Cooperative and cohesive interactions

Each individual i has two evolvable traits: (i) a 'cooperative tendency' modelled as a binary variable, denoted by $\omega_{c,i}$. Individuals with $\omega_{c,i} = 1$ always cooperate and those with $\omega_{c,i} = 0$ always defect. (ii) The second trait is a continuous variable 'cohesive tendency', denoted by $\omega_{s,i} \in [0, \infty)$. Individuals exhibit collective movement because of this cohesive tendency.

S1.3 Organismal mobility and cohesive interactions

To model organismal mobility, we consider two extreme scenarios:

1. An 'active' scenario modelling self-propelled individuals, such as birds, fish, mammals and flagellated microbes. Here, the medium has no influence on organismal movement and individuals actively display 'local flocking interactions' (attraction towards, and alignment with the direction of motion of their neighbours). Here, the cohesive tendency is related to the distance up to which an individual looks for neighbours to flock with. We call this distance as the 'local flocking radius' $R_{s,i}$. The cohesive tendency is then defined as $\omega_{s,i} = R_{s,i} - R_r$, where R_r is a measure of the body size or personal space of the individual, as will be described in the next section.

2. A 'passive' scenario, where individuals show no active movement, but are propelled by the medium that they live in, as in non-swimming bacteria or other microbes where the fluidity of the medium dominates individual motility. Here, the cohesive tendency is related to stickiness properties between individuals when they are in close contact. The cohesive tendency is the stickiness of the particles. We denote the stickiness by γ_i to avoid potential confusion with the slightly different definition of cohesive interactions in the self-propelled particle model.

S1.4 Model for movement of active (self-propelled) particles

All individuals move with a constant speed s. At each time step, individuals may modify their direction of motion due to interactions with their neighbours. The desired direction of motion $\boldsymbol{d}_i(t + \Delta t)$ at each time step is calculated according to the following movement rules. In addition, individuals' direction of motion is influenced by a small amount of stochasticity.

Note: Vectors denoted by $\boldsymbol{d}(t)$ are directions and thus always normalized after they are calculated.

1) **Repulsion**: The focal individual i moves away from individuals present within a distance R_r from itself, in the direction:

$$\boldsymbol{d}_{r,i}(t + \Delta t) = -\sum_{d_{ij} < R_r} \frac{\boldsymbol{c}_j(t) - \boldsymbol{c}_i(t)}{|\boldsymbol{c}_j(t) - \boldsymbol{c}_i(t)|} \tag{S1.1}$$

where $\boldsymbol{c}_i(t)$ and $\boldsymbol{c}_j(t)$ are the position vectors of individuals i and j respectively, and d_{ij} is the distance between them. This tendency for repulsion at short distances may be thought of as tendency of individuals to avoid collision with one another and that R_r could represent individuals' body size. Moving away from individuals within R_r takes precedence over all other movement decisions, so the next step (attraction and alignment) is skipped.

2) **Attraction and Alignment**: If there are no individuals within R_r of the focal individual, but there are individuals within a distance of $R_{s,i}\,(\geq R_r)$, the focal individual will exhibit local flocking interactions with its neighbours. The direction of motion due to these local interactions is a weighted average of the direction of attraction towards neighbours ($\boldsymbol{d}_{a,i}$) and the direction of alignment with their direction of motion ($\boldsymbol{d}_{o,i}$), calculated as follows:

$$\boldsymbol{d}_{a,i}(t) = \sum_{R_r<d_{ij}<R_s} \frac{\boldsymbol{c}_j(t) - \boldsymbol{c}_i(t)}{|\boldsymbol{c}_j(t) - \boldsymbol{c}_i(t)|}$$
$$\boldsymbol{d}_{o,i}(t) = \sum_{R_r<d_{ij}<R_s} \frac{\boldsymbol{v}_j(t)}{|\boldsymbol{v}_j(t)|} \tag{S1.2}$$

where $\boldsymbol{v}_j(t)$ is the velocity of individual j. The desired direction of motion is then calculated as follows:

$$\boldsymbol{d}_{s,i}(t+\Delta t) = k_a \boldsymbol{d}_{a,i}(t) + k_o \boldsymbol{d}_{o,i}(t) + (1 - k_a - k_o)\boldsymbol{d}_{s,i}(t) \tag{S1.3}$$

If there are no other individuals within a distance $R_{s,i}$, then both $\boldsymbol{d}_{a,i}(t)$ and $\boldsymbol{d}_{o,i}(t)$ are zero, and the desired direction is the same as the previous direction, with some added error as described in the next point. The parameters k_a and k_o represent the tendencies to attract and align with neighbours respectively, such that $k_a + k_o \leq 1$ and are chosen based on the empirical work of [2].

3) **Constraints**: Once the desired direction of motion is computed, two constraints are imposed to make the movement rules more realistic. i) Copying error: Since individuals may make mistakes in copying the directions and velocities of other individuals, a small vector error $\boldsymbol{\eta}_{ce}$ is added to the desired direction. Each component of this vector error is normally distributed with mean zero and variance σ_{ce}^2. ii) Turning rate constraint: An individual cannot turn instantaneously, but at a maximum rate ω_{max}. The maximum angle that it can turn in a single time step is then $\theta_{max} = \omega_{max}\Delta t$. The final direction taken is then $\boldsymbol{d}_i(t+\Delta t) = \text{turn}(\boldsymbol{d}_{s,i}(t+\Delta t) + \boldsymbol{\eta}_{ce})$ (OR $\text{turn}(\boldsymbol{d}_{r,i}(t+\Delta t) + \boldsymbol{\eta}_{ce})$, in case of repulsion). Here turn() means the vector is turned towards the desired direction up to maximum turning of θ_{max}.

The position in the next step is then calculated as

$$\boldsymbol{c}_i(t+\Delta t) = \boldsymbol{c}_i(t) + s\Delta t \boldsymbol{d}_i(t+\Delta t) \tag{S1.4}$$

We fix the values of k_a and k_o throughout the simulation, but evolve the local flocking tendency (R_s) across generations based on pay-off structures described in sections S1.6 and S5.1. Different values of R_s give rise to various types of movement, like solitary movement at $R_s \leq R_r$, fission-fusion grouping at medium values of R_s and large groups at very high R_s. Since $R_{s,i}$ is the evolvable cohesive trait, we are not imposing the nature of collective movement in our simulations.

S1.5 Model for movement of passive particles

Passive individuals (or particles) are completely driven by the medium. An individual will stick to the other individual with a straight γ_i when it comes in contact others. We modify the above movement model and adapt it to simulate this behaviour. To model the medium, we simulate the velocity field of a turbulent fluid with various levels of turbulence, following [3].

S1.5.1 A synthetic turbulence model

We model a two-dimensional, isotropic, homogenous fluid flow with zero mean velocity. We assume that the flow is divergence-free (as there are no fluid sources in the area of interest), and can therefore be represented by a potential function, ψ. The streamlines of the velocity field then $\mathbf{v}$ follow the contour lines of ψ, as

$$\mathbf{v} = (\frac{\partial \psi}{\partial y}, -\frac{\partial \psi}{\partial x}) \tag{S1.5}$$

The potential function ψ, is assumed to follow the following stochastic partial differential equation:

$$\frac{\partial \psi}{\partial t} = \nu \nabla^2 \psi + \sqrt{\xi} \frac{\partial W}{\partial t} \tag{S1.6}$$

with the following features: The diffusion coefficient ν determines the time scale of the fluid flow reaching an equilibrium, ξ defines the strength of stochastic fluctuations in the flow, (i.e. higher the value of ξ, more turbulent the fluid becomes), and W is a coloured noise with energy at 2-D frequency $k = (k_x, k_y)$ given by $\lambda_k = \lambda_0 e^{-\mu |k|}$. If we expand W as a Fourier series,

$$W(x/L, t) = \sum_{k \in 2\pi \mathbb{Z}^2} \sqrt{\lambda_k} \hat{W}_k(t) e^{ikx/L} \tag{S1.7}$$

Substituting this expansion in Eq (S1.6) leads to a system of Ornstein-Uhlenbeck equations:

$$d\hat{\psi}_k = -\nu |k|^2 \hat{\psi}_k dt + \sqrt{\xi \lambda_k} d\hat{W}_k \tag{S1.8}$$

which can then be evolved in Fourier space exactly using the theory of stochastic integration [4]:

$$\hat{\psi}_k(t+\Delta t) = \hat{\psi}_k(t)e^{-\nu|k|^2\Delta t} + \sqrt{\frac{\xi\lambda_k}{2\nu|k|^2}(1-e^{-2\nu|k|^2\Delta t})}\; Z_k \tag{S1.9}$$

where Z_k are random numbers sampled from the $N(0,1)$, with the constraint that $Z_k = Z^*_{-k}$, so that ψ is real valued.

We solve these set of equations by initializing ψ as a delta function, so that $\hat{\psi}(k) = 1 \; \forall \; k$.

S1.5.2 Passive (tracer) particles in fluid medium

We assume that the inertia of particles can be ignored and thus, are carried by the flow along its streamlines. The movement and cohesive interactions among particles is implemented in a simple way by modifying the movement model for active particles.

If two individuals are within a distance R_r of each other, they repel from each other and their desired direction of motion given by

$$\boldsymbol{d}_i(t+\Delta t) = -\sum_{d_{ij}<R_r} \frac{\boldsymbol{c}_j(t) - \boldsymbol{c}_i(t)}{|\boldsymbol{c}_j(t) - \boldsymbol{c}_i(t)|} \tag{S1.10}$$

where the summation is over all individuals within this short distance of repulsion.

If there are no individuals within the short repulsion area, individuals will be attracted towards neighbors who are within a distance of R_s. In such a case, the desired direction motion is given by

$$\boldsymbol{d}_i(t+\Delta t) = \sum_{R_r<d_{ij}<R_s} \frac{\boldsymbol{c}_j(t) - \boldsymbol{c}_i(t)}{|\boldsymbol{c}_j(t) - \boldsymbol{c}_i(t)|} + \boldsymbol{\eta}_{ce} \tag{S1.11}$$

where $\boldsymbol{\eta}_{ce}$ represents a random vector with mean zero and variance σ^2_{ce}.

The final speed and the direction of motion of the particle is then given by

$$\boldsymbol{v}_i(t+\Delta t) = \boldsymbol{v}_f(t) + \gamma_i \boldsymbol{d}_i(t) \tag{S1.12}$$

where $\boldsymbol{v}_f(t)$ is the flow velocity and γ_i is the strength of cohesive interactions arising from stickiness or adhesive property of the individual i. Note that we do add a small noise to the individual's desired direction of the motion but due to lack of interim of individuals, no turning rate constraint is applied. This model

can capture a range of behaviours where individuals trace the fluid when solitary, but stick together and move as a group when they come very close. We also note that the evolvable trait in this model is γ_i and we fix the radius of interaction to a constant.

S1.6 Payoff structure

Individuals move according to the movement rules (of either the active or passive system) for n_m steps. Depending on the strength of cohesive interactions, individuals may be found in groups. We define any two individuals as belonging to the same group if they are within a distance R_g from each other. We identify groups using a standard union-find algorithm [5]. We assume that individuals perform cooperative interactions with other individuals within their group at the n_m^{th} time step.

In the active system, if a group g has n_g individuals of which k_g are cooperators, then all cooperators in that group receive a payoff of

$$V_c(g) = \frac{k_g - 1}{n_g - 1} b - c - c_s R_{s,i}^2 \tag{S1.13}$$

whereas all defectors of that group receive a payoff of

$$V_d(g) = \frac{k_g}{n_g - 1} b - c_s R_{s,i}^2 \tag{S1.14}$$

where b is the benefit received from cooperators in the group (excluding self), c is the cost of cooperation and $c_s R_{s,i}^2 (= c_s (R_r + \omega_{s,i})^2)$ is the cost of cohesive interactions. Thus, in the active system, increasing the cost of cohesion also effectively increases baseline fitness by $c_s R_r^2$. Note that solitary cooperators get a payoff of $-c$, and solitary defectors get zero payoff. We discuss the derivation of this payoff structure and its modifications in section S5.1.

In the passive system, $R_{s,i}$ in the above equations is replaced with γ_i.

The fitness of each individual is a certain baseline value (V_0) plus payoffs arising from cooperative and flocking interactions:

$$V_i = V_0 + V_{c/d,i} - \min_i (V_{c/d,i}) \tag{S1.15}$$

We subtract $\min_i(V_{c/d,i})$ so that even the least fit individual has a positive fitness V_0. This allows us to control the strength of selection by varying the value of V_0. A low value corresponds to strong selection, and a high value to weak selection.

S1.7 Reproduction and dispersal

Individuals compete globally for reproduction, i.e. they reproduce with probability of reproduction proportional to their relative fitness in the entire population. Reproduction is asexual and synchronous. A roulette-wheel algorithm is used to generate N offspring for the next generation. All parents are immediately removed from the system after offspring are created. When an individual reproduces, it passes on it's two traits ($\omega_{s,i}$ and $\omega_{c,i}$) with a small mutation rate to its offspring. For the trait $\omega_{s,i}$, the mutation is implemented as an addition of a normally distributed noise $\eta_{\mu s}$ with mean zero and standard deviation $\sigma_{\mu s}$. For the binary cooperative trait, $\omega_{c,i}$ is flipped with a probability $p_{\mu c}$. The offspring are dispersed to random locations in space and with random orientation.

S1.8 Simulation initialisation and replicates

Simulations start with individuals located at random positions with random directions of motion. At the beginning of the first generation, all individuals have $\omega_{s,i} = 0$ (i.e. have no cohesive tendency) and $\omega_{c,i} = 0$ (all are defectors). A single simulation consists of T generations, with each generation consisting of n_m movement steps followed by cooperative interactions, reproduction, death of parent, and dispersal of offspring. Each simulation is replicated n_{rep} times with different initial positions, initial directions and seeds for the random number generators.

For the results presented in the main text and parameter scans in the supplementary information, we simulated 10 replicates, each consisting of 2000 generations of 1024 individuals, each generation with 2000 movement steps, unless otherwise stated (simulations for the analyses of timeseries were run until 32000 generations). The number of particles that can be simultaneously simulated presents a strong constraint on the simulation capacity. For the results produced according to the above scheme, a single data point required simulation of 2000 steps/generation $\times$ 2000 generations per simulation $\times$ 10 replicates $= 4 \times 10^7$ movement steps per individual, or 40-billion movement steps per data point.

Our simulations were written in CUDA-C++ and run on an Nvidia Tesla-K20 GPU with CUDA 5.5. With this configuration, the time taken to simulate 32 independent parallel runs up to 2000 generations was 2 hours (i.e. 20 hours for all

10 replicates). The entire code is available on GitHub at the following URLs: `https://github.com/tee-lab/altruism_active`, and `https://github.com/tee-lab/altruism_passive`.

S1.9 Instantaneous, average and evolved trait values

In each simulation, we keep track of the two evolvable variables of all individuals in the population: local cohesive tendencies $\omega_{s,i}$ and their corresponding cooperative trait (cooperator or defector). We do often plot instantaneous population averages of these quantities, the instantaneous average cohesive tendency of the population (ω_s) and the instantaneous proportion of cooperators in the population (p). Thus,

$$p = \frac{1}{N}\sum_{i=0}^{N}\omega_{c,i} \tag{S1.16}$$

$$\omega_s = \frac{1}{N}\sum_{i=0}^{N}\omega_{s,i} \tag{S1.17}$$

Furthermore, the average cohesive tendency of cooperators and defectors is calculated as follows:

$$\omega_{sc} = \frac{1}{pN}\sum_{i=0}^{N}\omega_{s,i}\omega_{c,i} \tag{S1.18}$$

$$\omega_{sd} = \frac{1}{(1-p)N}\sum_{i=0}^{N}\omega_{s,i}(1-\omega_{c,i}) \tag{S1.19}$$

The 'evolved values' of p and ω_s are obtained by averaging p and ω_s over all generations in a simulation (leaving out the first $T_{\text{trans}} = 500$ generations to allow transients to die down), and again averaging over the n_{rep} replicates.

$$\bar{p} = \frac{\sum_{i=0}^{n_{rep}}\sum_{t=T_{\text{trans}}}^{T} p}{n_{rep}T} \tag{S1.20}$$

$$\bar{\omega}_s = \frac{\sum_{i=0}^{n_{rep}}\sum_{t=T_{\text{trans}}}^{T} \omega_s}{n_{rep}T} \tag{S1.21}$$

These evolved values are then plotted against various parameter values.

**Parameter/constant**	**Symbol**	**Value**
**System properties**		
Number of individuals	N	1024
System size	L	300
Radius of grouping	R_g	2
Movement time step	Δt	0.2
**Individual traits**		
Radius of repulsion	R_r	1
Radius of flocking interactions	R_s	evolvable
Cooperative tendency	ω_c	evolvable
Cohesive tendency	ω_s	evolvable
**Movement traits**		
Coefficient of attraction	k_a	0.4
Coefficient of alignment	k_o	0.4
Maximum turning rate	ω_{max}	50°/time unit
Error in copying direction	$\boldsymbol{\eta}_{ce}$	-
SD of error in copying direction	σ_{ce}	0.05
Speed	s	1
Movement steps per generation	n_m	2000
**Selection**		
Benefit from cooperators	b	100
Cost of cooperation	c	...
Cost of cohesion	c_s	...
Fitness	V	-
Baseline fitness	V_0	1
Number of individuals in a group g	n_g	-
Number of cooperators in a group g	k_g	-
Probability of mutation of coop. tendency	$p_{\mu c}$	0.005
SD of mutation in cohesive tendency	$\sigma_{\mu s}$	0.1
Generations per run	T	2000
**Quantification**		
Number of replicates	n_{rep}	10
Measure of assortment	r	-
**Turbulence model**		
Diffusion constant of fluid flow	ν	0.005
Strength of stochastic fluctuations in fluid flow	ξ	0.05
Velocity of turbulent medium	v_f	-
Turbulence parameter	μ	...
Fluid energy parameter	λ_0	...
Particle stickiness	γ	evolvable

Table A: Symbols and parameter values. These values are used in all plots, unless specified otherwise. Parameters typically varied in plots marked by ..., dynamic quantities marked by -

References

[1] Guttal V, Couzin ID. Social interactions, information use, and the evolution of collective migration. Proceedings of the National Academy of Sciences. 2010;107(37):16172–16177. doi:10.1073/pnas.1006874107.

[2] Ioannou CC, Guttal V, Couzin ID. Predatory Fish Select for Coordinated Collective Motion in Virtual Prey. Science. 2012;337(6099):1212–1215. doi:10.1126/science.1218919.

[3] Torney C, Neufeld Z, Couzin ID, Levin SA. Context-Dependent Interaction Leads to Emergent Search Behavior in Social Aggregates. Proceedings of the National Academy of Sciences of the United States of America. 2009;106(52):22055–22060. doi:10.1073/pnas.0907929106.

[4] Gardiner CW. Handbook of stochastic methods. vol. 4. Springer Berlin; 1985.

[5] Cormen TH. Introduction to algorithms. MIT press; 2009.

[6] Wilson DS. A theory of group selection. Proceedings of the National Academy of Sciences. 1975;72(1):143–146.

[7] PEPPER JW. Relatedness in Trait Group Models of Social Evolution. Journal of Theoretical Biology. 2000;206(3):355 – 368. doi:http://dx.doi.org/10.1006/jtbi.2000.2132.

[8] Axelrod R, Hamilton WD. The evolution of cooperation. Science. 1981;211(4489):1390–1396. doi:10.1126/science.7466396.

[9] McElreath R, Boyd R. Mathematical models of social evolution: A guide for the perplexed. University of Chicago Press; 2008.
