# Supplementary material for: Mobility can promote the evolution of cooperation via emergent self-assortment dynamics
Source: PLoS Comput Biol. 2017 Sep 8;13(9):e1005732. doi: 10.1371/journal.pcbi.1005732 (PMC5607214; doi:10.1371/journal.pcbi.1005732)
Supplement: S2 Appendix — (PDF) [file pcbi.1005732.s002.pdf]

S2 Appendix Robustness to parameters

Changing the various parameter values does not alter the qualitative features of the model. Figures below show the effects of different parameter values on evolved $\bar{p}$ and $\bar{R}_s$ (these scans have been carried out for the active case only. Similar results are expected for the passive case). A common feature that emerges from these scans is that individuals seem to evolve such a value of R_s that allows them to form groups of a sufficient size.

S2.1 Effect of movement parameters

We chose a movement model that can exhibit a range of movement types by varying a single parameter (namely, ω_s). Thus, the model shows solitary movement for $\omega_s = 0$, fission-fusion groups of intermediate size for medium values of ω_s, and a few large groups for very high values of ω_s. As long as the movement model satisfies this condition, the particular choice of the model is not expected to significantly alter the results. Our results are robust to variations in the movement parameters, such as the attraction and orientation coefficients, and speed of movement, as discussed below.

First, we test the effect of attraction and orientation paramenters (Fig S1A-B). When k_a is very high, individuals tend to swarm around within a small locality and the group as a whole does not move much. Hence higher R_s (recall that $R_s = \omega_s + R_r$ for the active case, R_r being constant) helps find neighbours over a larger radius. On the other hand when k_o is high, individuals migrate a lot and there is more scope for new encounters. Here, larger R_s is not required.

S2.2 Effect of system size and population density

Similarly, when density is high, encounters are more likely (even by chance) and hence, a high flocking tendency is not required to form groups. Hence we find low R_s for low arena-size (Fig S1C-D show the effect of system size on the evolved values).

A third variable that gives the same inference is the grouping radius R_g. With high R_g larger groups can be formed even with less flocking, whereas with smaller R_g, more flocking (high R_s) is necessary to form larger groups (See Fig S1E-F).

S2.3 Effect of turbulence in the fluid medium

Finally, we also test robustness to the main parameter of the turbulence model. This parameter (μ) controls the frequency content of the fluid velocity field. Lower values of μ correspond to slower decay of energy along the frequency axis, in turn causing higher levels of turbulence. Higher values of μ correspond to lower turbulence. We keep the fluid velocity approximately constant while changing μ. We find that evolved proportion of cooperators remains more or less constant, but the stickiness evolves to lower values with decreasing turbulence. Again, this is because as the flow becomes more streamlined, groups are able to remain stable with lower values of stickiness. (Fig S1G)

In other words, we can say that R_s evolves to such a value as to maximize the benefit of cooperation. Hence we see that even though the evolved $\bar{R}_s$ shows a significant effect of parameters, the evolved $\bar{p}$ does not change significantly, and remains more or less close to the maximum value allowed by the cost of cooperation and cyclical dynamics.

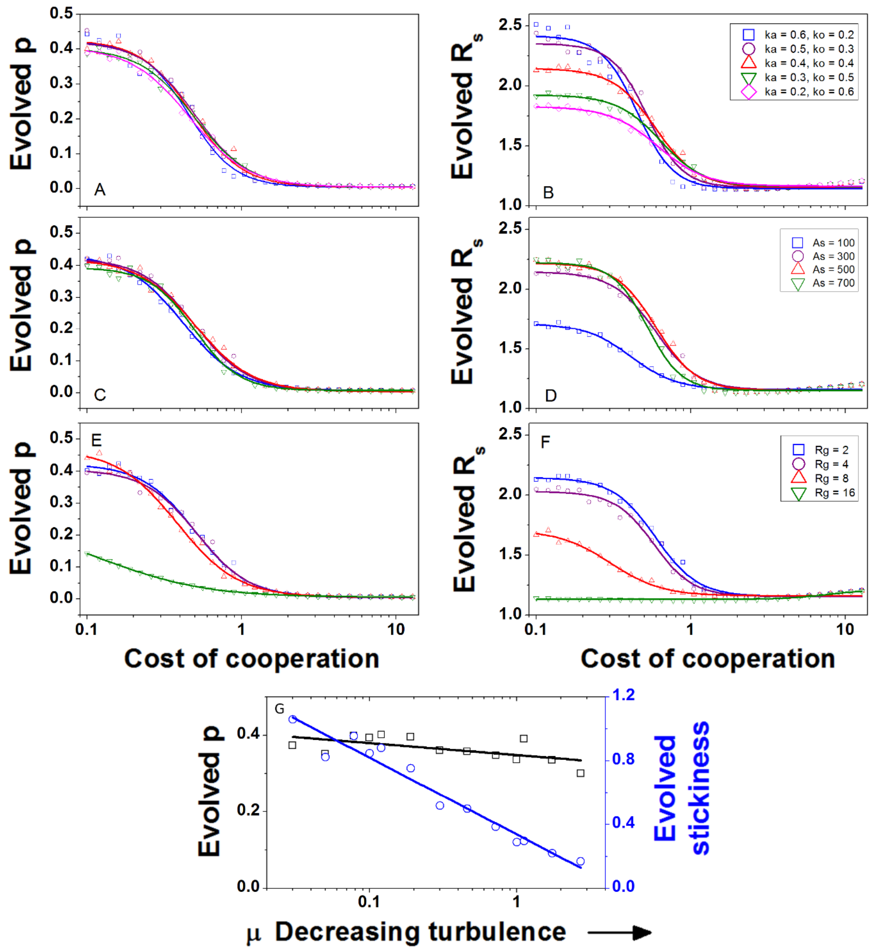

Figure S 1: Parameter Scans. Left column has evolved proportion of cooperators vs. cost of cooperation for different parameter values, right column has evolved flocking tendency vs. cost of cooperation for different parameter values. Each row corresponds to one parameter that changes while all others are held fixed. Parameters: $c_s = 2$. Other parameters as in Table A (see Methods). In (G), μ and λ_0 were varied together to produce approximately constant average fluid velocity of 1 unit.

References

[1] Guttal V, Couzin ID. Social interactions, information use, and the evolution of collective migration. Proceedings of the National Academy of Sciences. 2010;107(37):16172–16177. doi:10.1073/pnas.1006874107.

[2] Ioannou CC, Guttal V, Couzin ID. Predatory Fish Select for Coordinated Collective Motion in Virtual Prey. Science. 2012;337(6099):1212–1215. doi:10.1126/science.1218919.

[3] Torney C, Neufeld Z, Couzin ID, Levin SA. Context-Dependent Interaction Leads to Emergent Search Behavior in Social Aggregates. Proceedings of the National Academy of Sciences of the United States of America. 2009;106(52):22055–22060. doi:10.1073/pnas.0907929106.

[4] Gardiner CW. Handbook of stochastic methods. vol. 4. Springer Berlin; 1985.

[5] Cormen TH. Introduction to algorithms. MIT press; 2009.

[6] Wilson DS. A theory of group selection. Proceedings of the National Academy of Sciences. 1975;72(1):143–146.

[7] PEPPER JW. Relatedness in Trait Group Models of Social Evolution. Journal of Theoretical Biology. 2000;206(3):355 – 368. doi:http://dx.doi.org/10.1006/jtbi.2000.2132.

[8] Axelrod R, Hamilton WD. The evolution of cooperation. Science. 1981;211(4489):1390–1396. doi:10.1126/science.7466396.

[9] McElreath R, Boyd R. Mathematical models of social evolution: A guide for the perplexed. University of Chicago Press; 2008.
