# Supplementary material for: Mobility can promote the evolution of cooperation via emergent self-assortment dynamics
Source: PLoS Comput Biol. 2017 Sep 8;13(9):e1005732. doi: 10.1371/journal.pcbi.1005732 (PMC5607214; doi:10.1371/journal.pcbi.1005732)
Supplement: S3 Appendix — (PDF) [file pcbi.1005732.s003.pdf]

S3 Appendix Analytical calculations for simple cases using selection mutation equilibrium

S3.1 Change in proportion of cooperators

Let p be the proportion of cooperators in the population. Let the population be divided into multiple interacting groups. Then, the payoff to each cooperator and defector will be based on its group composition. Further, let the average payoff to cooperators and defectors be V_c and V_d. Then average fitness of the population is

$$\bar{V} = pV_c + (1-p)V_d \tag{S3.1}$$

We assume that after all individuals have received payoffs from interactions within their respective groups, they reproduce synchronously, with fecundity proportional to their payoffs. Further, we also assume that the population size is constant and competition is global. Therefore, it follows by definition, that, in the next generation, the proportions of cooperators (p') and defectors ($1-p'$) would be

$$\begin{aligned} p' &= \frac{pV_c}{\bar{V}} \\ 1-p' &= \frac{(1-p)V_d}{\bar{V}} \end{aligned} \tag{S3.2}$$

Out of the p' new cooperators, a fraction μ will mutate into defectors, and similarly, from the $1-p'$ new defectors, a fraction μ will mutate into cooperators. Since the offsspring disperse randomly, mutants are equally likely to appear anywhere in space. The new frequency of cooperators after mutation would therefore be

$$p'' = \frac{pV_c(1-\mu)}{\bar{V}} + \frac{\mu(1-p)V_d}{\bar{V}} \tag{S3.3}$$

The change in frequency of cooperators over the previous generation would be

$$
\begin{aligned}
\Delta p &= p'' - p \\
&= \frac{pV_c(1-\mu)}{\bar{V}} + \frac{\mu(1-p)V_d}{\bar{V}} - p \\
&= \frac{1}{\bar{V}}\Big(pV_c(1-\mu) + \mu(1-p)V_d - p(pV_c + (1-p)V_d)\Big) \\
&= \frac{1}{\bar{V}}\Big(pV_c - \mu pV_c + \mu(1-p)V_d - p^2V_c + p(1-p)V_d\Big) \\
&= \frac{1}{\bar{V}}\Big(p(1-p)(V_c - V_d) - \mu(pV_c - (1-p)V_d\Big) \\
&= \frac{p(1-p)(V_c - V_d)}{\bar{V}} - \mu\Big(\frac{pV_c - (1-p)V_d}{\bar{V}}\Big)
\end{aligned}
\tag{S3.4}
$$

S3.2 Selection-mutation equilibrium

In equilibrium, we can calculate p by setting $\Delta p = 0$. We now use this equation to analytically calculate p for some simple cases.

S3.3 Baseline Scenarios

We compare the results of our coevolutionary simulations with various baseline scenarios. In any baseline scenario, only one of the traits (ω_c or ω_s) is allowed to evolve, while holding the other trait constant at some value. We consider three baseline scenarios here, for two of which, we derive the expected proportion of cooperators analytically.

S3.3.1 All solitary individuals

The simplest baseline case is where every individual is solitary, and the population is large enough that effects of drift are negligible. Then, according to our payoff scheme, $V_c = 1$ and $V_d = 1 + c$. Substituting in equation (S3.4), we get

$$
p = \frac{\mu(c+2) + c - \sqrt{c^2(\mu-1)^2 + 4\mu^2(1+c)}}{2c} \tag{S3.5}
$$

The maximum value of p in this case is ≈ 0.05, much lower than the coevolved value, and decreases with increasing cost. We verified this result with simulation as well.

S3.3.2 Single large group

Naturally, being a cooperator does not pay off when everyone else is solitary. The other extreme where cooperators cannot sustain invasion by defectors is when the whole population forms a single, well-mixed group. This can be simulated by setting a very high value of R_s, so that all individuals form a single large group. In this case, for large N, $V_c = V_0+pb-c-\frac{b}{N} \approx V_0+pb-c$ and $V_d = V_0+pb$. Since the minimum fitness in the population at any generation would be $V_{min} = V_0+pb-c$, the relative fitnesses according to our scheme would again be $V_c = 1$ and $V_d = 1+c$, giving the same value of p as above.

S3.3.3 No cohesion

When ω_s is set to zero, individuals perform no cohesive interactions, and thus their movement is a random walk. However, if two or more individuals happen to be in close proximity just by chance, then they can still perform cooperative interactions, resulting in slightly higher proportion of cooperators than in the above baselines.

S3.4 Semi-analytical calculation of expected p in the co-evolutionary case

It is possible to quantify the effect of self-sorting using a semi-analytical, heuristic approach. We find from the simulations that across generations the average value of positive assortment is zero. This is expected, because in the complete cycle, ΔR_s is positive during one part of the cycle and negative in the other part, so that average $\Delta R_s = 0$. We calculate the expected proportion of cooperators assuming that the population is in simple equilibrium, without any cyclicity. In such a case, p would be constant. Also since $\Delta R_s = r = 0$ on average, the difference in fitness of cooperators and defectors would be $\Delta V = rb - c = -c$. Now with the equilibrium p and R_s, the population would consist of dynamic groups each with p_g proportion of cooperators, and the average fitness of any such group would be $V_g = V_0+p_g(b-c)$. Since r is zero on average, the average fitness of the population is $\bar{V} = E_g[V_g] = V_0 + p(b-c)$. Furthermore, for small enough c, we assume that $\bar{V} \gg c$, so that $V_c \approx V_d \approx \bar{V}$. Plugging these values in equation (S3.4), we get

$$\begin{aligned} \bar{V}\Delta p &= p(1-p)(rb-c) - \mu(2p-1)\bar{V} = 0 \\ \implies & p(1-p)(-c) - \mu(2p-1)\bar{V} = 0 \end{aligned} \tag{S3.6}$$

Solving, we get

$$p = \frac{-\mu(b-c) + c + 2\mu V_0 - \sqrt{(\mu(b-c) - c - 2\mu V_0)^2 - 4\mu V_0(-2\mu(b-c) + c)}}{2(-2\mu(b-c) + c)} \tag{S3.7}$$

Using this semi-analytical equation, we can calculate the expected proportion of cooperators for different mutation rates and compare it with that observed (Fig S2H).

References

[1] Guttal V, Couzin ID. Social interactions, information use, and the evolution of collective migration. Proceedings of the National Academy of Sciences. 2010;107(37):16172–16177. doi:10.1073/pnas.1006874107.

[2] Ioannou CC, Guttal V, Couzin ID. Predatory Fish Select for Coordinated Collective Motion in Virtual Prey. Science. 2012;337(6099):1212–1215. doi:10.1126/science.1218919.

[3] Torney C, Neufeld Z, Couzin ID, Levin SA. Context-Dependent Interaction Leads to Emergent Search Behavior in Social Aggregates. Proceedings of the National Academy of Sciences of the United States of America. 2009;106(52):22055–22060. doi:10.1073/pnas.0907929106.

[4] Gardiner CW. Handbook of stochastic methods. vol. 4. Springer Berlin; 1985.

[5] Cormen TH. Introduction to algorithms. MIT press; 2009.

[6] Wilson DS. A theory of group selection. Proceedings of the National Academy of Sciences. 1975;72(1):143–146.

[7] PEPPER JW. Relatedness in Trait Group Models of Social Evolution. Journal of Theoretical Biology. 2000;206(3):355 – 368. doi:http://dx.doi.org/10.1006/jtbi.2000.2132.

[8] Axelrod R, Hamilton WD. The evolution of cooperation. Science. 1981;211(4489):1390–1396. doi:10.1126/science.7466396.

[9] McElreath R, Boyd R. Mathematical models of social evolution: A guide for the perplexed. University of Chicago Press; 2008.
