# Supplementary material for: Mobility can promote the evolution of cooperation via emergent self-assortment dynamics
Source: PLoS Comput Biol. 2017 Sep 8;13(9):e1005732. doi: 10.1371/journal.pcbi.1005732 (PMC5607214; doi:10.1371/journal.pcbi.1005732)
Supplement: S4 Appendix — (PDF) [file pcbi.1005732.s004.pdf]

S4 Appendix Deriving a measure of assortment

We first derive the condition for increase in cooperators using first principles. Then, we compare this condition with that predicted by the Price Equation. We find that the condition derived using Price Equation is identical to that derived using first principles, demonstrating that the Price Equation applies to our problem. We then use the idea of relatedness from the inclusive fitness theory to derive a measure of assortment of the cooperative genotype. We also show that we get the same condition for increase in cooperators using the multilevel selection approach.

S4.1 Condition for increase in cooperators

Suppose that in a given generation, the population is divided into G interacting subgroups. Let the number of cooperators in group g be k_g and the size of group g be n_g.

Then the average fitness of cooperators is given by

$$\begin{aligned} V_c &= V_0 + \frac{1}{pN}\sum_{g=1}^{G}\left(\frac{k_g-1}{n_g-1}b - c\right)k_g \\ &= V_0 + \frac{1}{pN}\sum_{g=1}^{G}\left(\frac{k_g^2}{n_g-1}b - \frac{k_g}{n_g-1}b - ck_g\right) \end{aligned} \tag{S4.1}$$

Similarly, the average fitness of defectors is

$$\begin{aligned} V_d &= V_0 + \frac{1}{(1-p)N}\sum_{g=1}^{G}\left(\frac{k_g}{n_g-1}b\right)(n_g-k_g) \\ &= V_0 + \frac{1}{(1-p)N}\sum_{g=1}^{G}\left(\frac{k_g n_g}{n_g-1}b - \frac{k_g^2}{n_g-1}b\right) \end{aligned} \tag{S4.2}$$

The difference in fitness is therefore

$$
\begin{aligned}
V_c - V_d &= \frac{1}{N}\sum_{g=1}^{G}\left(\frac{1}{p(1-p)}\frac{k_g^2}{n_g-1}b - \frac{1}{p}\frac{k_g}{n_g-1}b - \frac{1}{1-p}\frac{k_g n_g}{n_g-1}b\right) - c \\
&= \frac{1}{Np(1-p)}\sum_{g=1}^{G}\left(\frac{k_g^2}{n_g-1}b - (1-p)\frac{k_g}{n_g-1}b - p\frac{k_g n_g}{n_g-1}b\right) - c \qquad \text{(S4.3)}
\end{aligned}
$$

Now, from Eq S3.4, the condition for increase in cooperators ($\Delta p > 0$) is that $V_c > V_d$, i.e.

$$
\frac{1}{Np(1-p)}\sum_{g=1}^{G}\left(\frac{k_g^2}{n_g-1} - (1-p)\frac{k_g}{n_g-1} - p\frac{k_g n_g}{n_g-1}\right) > \frac{c}{b} \qquad \text{(S4.4)}
$$

This result is identical to that derived by D. S. Wilson [6]. We have neglected the effect of mutations in the calculations above, assuming that mutation rate is small.

The change in proportion of cooperators due to mutations (see Eq S3.4) is

$$
\begin{aligned}
\bar{V}\Delta p|_{\mu} &= -\mu(pV_c - (1-p)V_d) \\
&= -\frac{\mu}{N}\sum_{g=1}^{G}\left(\left(V_0 + \frac{k_g-1}{n_g-1}b - c\right)k_g - \left(V_0 + \frac{k_g-1}{n_g}b\right)(n_g - k_g)\right) \\
&= -\mu(V_0(2p-1) + br_{\mu} - cp) \qquad \text{(S4.5)}
\end{aligned}
$$

where

$$
r_{\mu} = \frac{1}{N}\sum_{g=1}^{G}\left(\frac{k_g}{n_g-1}(2k_g - n_g) - \frac{k_g}{n_g-1}\right) \qquad \text{(S4.6)}
$$

S4.2 Assortment of the cooperative genotype

The interactions within each generation are defined by the group structure of the population, which can be captured by a spatial snapshot of the system. We derive the assortment of genotypes (or the covariance of the actor's genotype on that of the recipients). We calculate assortment from spatial snapshots in our simulations.

Let p_{ig} be the indicator variable for individual i in group g ($p_{ig} = 1$ if ig is a cooperator, $p_{ig} = 0$ if ig is a defector), V_{ig} be the payoff (fecundity) of ig, and ω_{ig} its fitness. We start with Price equation

$$\bar{\omega}\Delta p = \mathrm{cov}(\omega_{ig}, p_{ig}) \tag{S4.7}$$

where $\bar{\omega}$ is the average population fitness, ω_{ig} is the fitness of individual i in group g. The covariance and expectation are taken across all individuals. Now since the population size is constant, $\bar{\omega} = 1$ and $\omega_g = V_g/\bar{V}$, where V_g is the group average payoff (or fecundity) and $\bar{V}$ is the population average payoff. Substituting in the above equation we get

$$\bar{V}\Delta p = \mathrm{cov}(V_{ig}, p_{ig}), \tag{S4.8}$$

where we have assumed that the transmission term (arising from mutations) is zero.

Now, the payoffs can be written as

$$\begin{aligned}
V_{ig} &= V_0 + \frac{k_g - p_{ig}}{n_g - 1} b - c p_{ig} && \text{(S4.9)}\\
&= V_0 + \frac{\sum_j p_{jg} - p_{ig}}{n_g - 1} b - c p_{ig} && \text{(S4.10)}\\
&= V_0 + \left(\frac{1}{n_g - 1} \sum_{j \neq i} p_{jg} \right) b - c p_{ig}, && \text{(S4.11)}
\end{aligned}$$

where $\frac{1}{n_g-1}\sum_{j\neq i} p_{jg}$ is the proportion of cooperators among co-players of the focal individual ig.

Substituting these payoffs into Price's equation and simplifying, we get

$$\begin{aligned}
\bar{V}\Delta p &= \mathrm{cov}_{ig}(V_{ig}, p_{ig}) && \text{(S4.12)}\\
&= b \cdot \mathrm{cov}\left(\frac{1}{n_g - 1} \sum_{j \neq i} p_{jg}, p_{ig} \right) - c \cdot \mathrm{cov}(p_{ig}, p_{ig}) && \text{(S4.13)}
\end{aligned}$$

Dividing both sides by $\mathrm{cov}(p_{ig}, p_{ig}) = \mathrm{var}(p_{ig})$, we then obtain that $\Delta p > 0$ if and only if $rb > c$, where

$$r = \frac{\mathrm{cov}_{ig}\left(\frac{1}{n_g-1}\sum_{j\neq i} p_{jg}, p_{ig}\right)}{\mathrm{var}_{ig}(p_{ig})} \tag{S4.14}$$

is a measure of assortment and is identical to the usual definition of relatedness given by the regression of the actor's genotype on the genotype of recipients [7].

We can simplify r further as follows:

$$\begin{aligned} r &= \frac{\mathrm{cov}\left(\frac{1}{n_g-1}\sum_{j\neq i} p_{jg},\ p_{ig}\right)}{\mathrm{var}(p_{ig})} \\ &= \frac{\mathrm{cov}\left(\frac{k_g - p_{ig}}{n_g-1}, p_{ig}\right)}{\mathrm{var}(p_{ig})} \end{aligned} \tag{S4.15}$$

Now,

$$\begin{aligned} E\left[\frac{k_g - p_{ig}}{n_g - 1}\right] &= \frac{1}{N}\sum_{g=1}^{G}\sum_{j=1}^{n_g}\left(\frac{k_g - p_{jg}}{n_g - 1}\right) \\ &= \frac{1}{N}\sum_g\left(\frac{k_g n_g}{n_g - 1} - \frac{k_g}{n_g - 1}\right) \\ &= p \end{aligned} \tag{S4.16}$$

Thus,

$$\begin{aligned} \mathrm{cov}\left(\frac{k_g - p_{ig}}{n_g - 1}, p_{ig}\right) &= \frac{1}{N}\sum_{g=1}^{G}\sum_{j=1}^{n_g}\left(\frac{k_g - p_{jg}}{n_g - 1} - p\right)(p_{ig} - p) \\ &= \frac{1}{N}\sum_g\left(\frac{k_g^2}{n_g - 1} - \frac{k_g}{n_g - 1} - p\frac{k_g n_g}{n_g - 1} + p\frac{k_g}{n_g - 1} - pk_g + p^2\right) \\ &= \frac{1}{N}\left(\sum_g \frac{k_g^2}{n_g - 1} - p\sum_g \frac{k_g n_g}{n_g - 1} - (1-p)\sum_g \frac{k_g}{n_g - 1}\right) \end{aligned} \tag{S4.17}$$

and

$$\mathrm{var}_{ig}(p_{ig}) = p(1-p), \tag{S4.18}$$

To account for solitary individuals, we split the summations in Eq S4.17 into groups with $n_g > 1$ and $n_g = 1$. The summations with $n_g = 1$, can be computed by adding a small ϵ to the denominator and letting it tend to zero:

$$\begin{aligned}
&\lim_{\epsilon\to 0}\frac{1}{N}\left(\sum_{n_g=1}\frac{k_g^2}{n_g-1+\epsilon} - p\sum_{n_g=1}\frac{k_g n_g}{n_g-1+\epsilon} - (1-p)\sum_{n_g=1}\frac{k_g}{n_g-1+\epsilon}\right)\\
&=\lim_{\epsilon\to 0}\frac{1}{N}\left(\sum_{n_g=1}\frac{k_g}{\epsilon} - p\sum_{n_g=1}\frac{k_g}{\epsilon} - (1-p)\sum_{n_g=1}\frac{k_g}{\epsilon}\right)\\
&=\lim_{\epsilon\to 0} 0\\
&=0
\end{aligned} \tag{S4.19}$$

Therefore, the assortment can be obtained by simply performing the sums in Eq S4.17 for groups with size greater than one.

The above assortment formula is the same as the threshold cost to benefit ratio calculated in Eq S4.4. This confirms that the Price equation applies to our system, and therefore, relatedness is an accurate measure of assortment.

S4.3 Multilevel selection analysis

Again, we start with the Price equation, and partition the covariance in a between-group component and a within-group component, to give the change in proportion of cooperators as follows:

$$\bar{V}\Delta p = \mathrm{cov}(V_g, p_g) + E_g[V_g \Delta p_g], \tag{S4.20}$$

We can further split the term $V_g\Delta p_g$ between and within individuals of group g as follows:

$$V_g\Delta p_g = \mathrm{cov}(V_{ig}, p_{ig}) + E[V_{ig}\Delta p_{ig}] \tag{S4.21}$$

where the second term is zero because we assume no mutations. Now,

$$\begin{aligned}
V_g \Delta p_g &= \text{cov}(V_{ig}, p_{ig}) \\
&= -c \ \text{var}(p_{ig}) - \frac{b}{n_g - 1}\text{var}(p_{ig}) \\
&= -p_g(1-p_g)\Big(\frac{b}{n_g - 1} + c\Big) \\
&= -p_g c + p_g^2 c + \frac{p_g^2}{n_g - 1} b - \frac{p_g}{n_g - 1} b
\end{aligned} \tag{S4.22}$$

and

$$E_g\left[V_g \Delta p_g\right] = -pc + cE\left[p_g^2\right] + bE\left[\frac{p_g^2}{n_g - 1}\right] - bE\left[\frac{p_g}{n_g - 1}\right] \tag{S4.23}$$

Also,

$$V_g = E_{ig}\left[V_i\right] = V_0 + (b-c)p_g \tag{S4.24}$$

and

$$\begin{aligned}
\text{cov}(V_g, p_g) &= (b-c) \ \text{var}(p_g) \\
&= (b-c)(E\left[p_g^2\right] - p^2) \\
&= -c(E\left[p_g^2\right] - p^2) + b\left(E\left[p_g^2 \frac{n_g - 1}{n_g - 1}\right] - pE\left[p_g \frac{n_g - 1}{n_g - 1}\right]\right) \\
&= -c(E\left[p_g^2\right] - p^2) \\
&\quad + b\left(E\left[\frac{p_g^2 n_g}{n_g - 1}\right] - E\left[\frac{p_g^2}{n_g - 1}\right] - pE\left[\frac{p_g n_g}{n_g - 1}\right] + pE\left[\frac{p_g}{n_g - 1}\right]\right)
\end{aligned} \tag{S4.25}$$

Adding Eq S4.22 and Eq S4.25, we get

$$
\begin{aligned}
\bar{V}\Delta p &= b\left(E\left[\frac{p_g^2 n_g}{n_g-1}\right] - pE\left[\frac{p_g n_g}{n_g-1}\right] - (1-p)E\left[\frac{p_g}{n_g-1}\right]\right) - cp(1-p) \\
&= \frac{b}{N}\left(\sum_g \frac{k_g^2}{n_g-1} - p\sum_g \frac{k_g n_g}{n_g-1} - (1-p)\sum_g \frac{k_g}{n_g-1}\right) - cp(1-p)
\end{aligned}
\tag{S4.26}
$$

which leads to the same condition $rb > c$ for increase in cooperator frequency.

References

[1] Guttal V, Couzin ID. Social interactions, information use, and the evolution of collective migration. Proceedings of the National Academy of Sciences. 2010;107(37):16172–16177. doi:10.1073/pnas.1006874107.

[2] Ioannou CC, Guttal V, Couzin ID. Predatory Fish Select for Coordinated Collective Motion in Virtual Prey. Science. 2012;337(6099):1212–1215. doi:10.1126/science.1218919.

[3] Torney C, Neufeld Z, Couzin ID, Levin SA. Context-Dependent Interaction Leads to Emergent Search Behavior in Social Aggregates. Proceedings of the National Academy of Sciences of the United States of America. 2009;106(52):22055–22060. doi:10.1073/pnas.0907929106.

[4] Gardiner CW. Handbook of stochastic methods. vol. 4. Springer Berlin; 1985.

[5] Cormen TH. Introduction to algorithms. MIT press; 2009.

[6] Wilson DS. A theory of group selection. Proceedings of the National Academy of Sciences. 1975;72(1):143–146.

[7] PEPPER JW. Relatedness in Trait Group Models of Social Evolution. Journal of Theoretical Biology. 2000;206(3):355 – 368. doi:http://dx.doi.org/10.1006/jtbi.2000.2132.

[8] Axelrod R, Hamilton WD. The evolution of cooperation. Science. 1981;211(4489):1390–1396. doi:10.1126/science.7466396.

[9] McElreath R, Boyd R. Mathematical models of social evolution: A guide for the perplexed. University of Chicago Press; 2008.
