# Supplementary material for: Mobility can promote the evolution of cooperation via emergent self-assortment dynamics
Source: PLoS Comput Biol. 2017 Sep 8;13(9):e1005732. doi: 10.1371/journal.pcbi.1005732 (PMC5607214; doi:10.1371/journal.pcbi.1005732)
Supplement: S5 Appendix — (PDF) [file pcbi.1005732.s005.pdf]

S5 Appendix Effect of model variations

S5.1 Effect of choice of payoff structure

Payoff due to cooperative interactions can be calculated in a variety of scenarios such as prisoner's dilemma game [8], sentinel behaviour, alarm calling, and public goods production. In our main result, we use the prisoner's dilemma game as described below, which presents the strictest constraints for the evolution of cooperation among these different payoff structures. To test the robustness of our result, we also consider payoffs associated with various other ecological contexts, and minor modifications to the prisoner's dilemma payoffs.

S5.1.1 Prisoner's dilemma payoff

Consider a group of n_g individuals out of which, k_g are cooperators. In the 'strong altruism' scenario, where the cooperators cannot benefit from their own act, each cooperator pays a cost c to produce a benefit b. This benefit is shared equally by the remaining $n_g - 1$ individuals. Conversely, a cooperator pays a cost c of its own cooperative act and benefits only from the cooperative acts of the other $k_g - 1$ cooperators. A defector benefits from the acts of all k_g cooperators, and does not pay any cost. Under these assumptions, the payoffs can be written as

$$\begin{aligned} V_c(g) &= \frac{k_g - 1}{n_g - 1} b - c \\ V_d(g) &= \frac{k_g}{n_g - 1} b \end{aligned} \tag{S5.1}$$

These payoffs can also be understood as a generalization of the pairwise Prisoner's Dilemma game [8] to n-players. In a pairwise game, when two cooperators interact, they both get a payoff of $b - c$, when two defectors interact, both get zero payoff, whereas when a cooperator and a defector interact, the defector gets payoff b and the cooperator gets $-c$ (Table B). We assume that the pairs form randomly, and do not depend on individuals' spatial positions within the groups. There are no repeated interactions. We calculate the average payoffs to cooperators and defectors under these assumptions.

Following the convention by McElreath and Boyd [9], if $P(A|B)$ is the probability of meeting an individual of type A, given that it is of type B, (where A and B

	**Prisoner's Dilemma**	Interacting with cooperator	Interacting with defector
Payoff to	cooperator	$b - c$	$-c$
Payoff to	defector	b	0

Table B: Payoff matrix for Prisoner's Dilemma game

could denote cooperator and defector respectively), and $V(B|A)$ is the payoff to individual of type B, having met an individual of type A, the payoffs are

$$\begin{aligned}
V_c(g) &= P(C|C)V(C|C) + P(D|C)V(C|D) \\
&= \frac{k_g - 1}{n_g - 1}(b - c) + \frac{n_g - k_g}{n_g - 1}(-c) \\
&= \frac{k_g - 1}{n_g - 1}b - c \\
V_d(g) &= P(C|D)V(D|C) + P(D|D)V(D|D) \\
&= \frac{k_g}{n_g - 1}b + \frac{n_g - k_g - 1}{n_g - 1} \times 0 \\
&= \frac{k_g}{n_g - 1}b
\end{aligned} \tag{S5.2}$$

We test the robustness of our simulations to various other cooperation scenarios such as sentinel behaviour and alarm calling, as well as minor modifications of the above payoff structure.

S5.1.2 Sentinel Behaviour payoff

In many grouping species, groups employ an individual as a sentinel to stand vigilant to watch out for predators and warn the others upon spotting one. The other individuals use this time for foraging, and are expected to stand guard once their turn arrives. If there are n_g individuals in the group, among which k_g are cooperators who actually stand guard during their turn, and $n_g - k_g$ individuals are defectors who cheat and do not stand guard in their turn (may even continue to forage). Let us divide the entire foraging duration into n_g time slots such that each individual stands as sentinel in 1 slot. Cooperative sentinels suffer the cost of increased predation risk as well as forgone foraging time, relative to cheaters.

Each cooperator then benefits during the sentinel slots of other cooperators, i.e for a fraction $\frac{k_g-1}{n_g}$ of the day, and pays a cost c/n_g (if c is assumed to be the cost paid per unit time of sentinel activity) during its own slot. Defectors on the other hand, benefit during the slots of all k_g cooperators, and do not pay any cost. Hence the payoffs to cooperators and defectors are:

$$\begin{aligned} V_c(g) &= \frac{k_g - 1}{n_g} b - \frac{c}{n_g} \\ V_d(g) &= \frac{k_g}{n_g} b \end{aligned} \tag{S5.3}$$

S5.1.3 Alarm calling payoff

Many animals show alarm calling behaviour, where there is no appointed sentinel, but each cooperator gives an alarm call upon spotting the predator. One way to model the payoffs in the case of alarm calling would be to assume that the benefits of the alarm call are not divided. Instead, all individuals get some benefit b from an alarm call independent of the group size. Furthermore, if we assume that the chances of spotting the predators (and thus the group getting benefited) increase linearly with the number of cooperators in the group, we arrive at the following payoffs.

$$\begin{aligned} V_c(g) &= (k_g - 1)b - c \\ V_d(g) &= k_g b \end{aligned} \tag{S5.4}$$

S5.1.4 Non-singular payoff

Another modification that we use in our analytical model is to replace $n_g - 1$ in the prisoner's dilemma payoffs by n_g. Here, we confirm that our main results are robust to this change (Fig S2C-D).

$$\begin{aligned} V_c(g) &= \frac{k_g - 1}{n_g} b - c \\ V_d(g) &= \frac{k_g}{n_g} b \end{aligned} \tag{S5.5}$$

S5.1.5 Conditional cooperation payoff

Previous studies have shown that cooperation can evolve in dynamic networks if individuals cooperate only when they are connected to a certain threshold number of other individuals. In our model, this corresponds to individuals cooperating only when they are in group larger than a certain threshold. The payoff to cooperators and defectors in large groups would be same are the prisoner's dilemma payoff, and all individuals in groups below that size would get zero payoff.

S5.1.6 Optimal group size payoff

In our main model, we assume that benefits of grouping can arise only from the synergistic effects of active cooperation. In the real world, the passive benefits of grouping such as many eyes effects can actually be large enough such that grouping is beneficial at small group sizes even without cooperation. Large groups however, remain costly, so that the payoff due to grouping peaks at an 'optimal' group size. We model the costs of grouping as

$$\text{cost of grouping} = -c_s R_s (R_s - R_{opt}) \quad \text{(S5.6)}$$

S5.1.7 Robustness to payoff structure

Fig S2C-D shows the proportion of cooperators for all the different payoff structures described above, compared with the Prisoners Dilemma payoffs used in the main simulations. As expected, minor variations in the payoffs do not drastically affect the result, except in the un-normalized payoff case. In this payoff scheme, the benefit to cooperators increases nonlinearly with the proportion of cooperators in the group, and hence an increase in p is expected.

S5.2 Effect of mobility and limited dispersal

As we argued in the main text, it is generally believed that mobility causes mixing and destroys positive assortment, but evolved differences in cohesiveness between individuals result in self sorting and maintain assortment in spite of fluidity in the population.

In the trivial case of zero mobility, we would have strictly local dispersal, and all groups would eventually be invaded by mutant defectors. If mobility is increased

slightly, local dispersal with occasional mixing permits cooperator groups to occasionally form, giving rise to a very high proportion of cooperators on average. We simulate this by starting with local dispersal and allowing movement for only 2000 steps until cooperative interactions happen, and find that as expected, the evolved proportion of cooperators is high (Fig S2A-B, 2k+LD). If mobility is very high, however, in spite of local dispersal, the population would become well mixed (with the small assortment caused by differences in cohesive tendencies). We simuate this by allowing 8000 movement steps after local dispersal, and find that the result is identical to our main coevolutionary case (Fig S2A-B, 8k+LD, 2k+RD, 8k+RD).

Let us now consider what happens in the case of random dispersal. Again, in the trivial case of zero mobility, all individuals would be solitary (after dispersal), and cooperation will not evolve. If we allow only slight mobility, then there is not enough time for self sorting to happen, and we will get small groups of random composition.

In summary, if dispersal is random, mobility promotes cooperation by allowing for self sorting of cooperators, as compared to random group formation following dispersal. If dispersal is local, then mobility hinders cooperation in spite of movement driven self sorting, as compared to when low mobility allows cooperator groups to remain intact over multiple generations.

S5.3 Effect of strength of selection

We change the strength of selection by changing the baseline fitness value. A Higher baseline fitness corresponds to weak selection. Qualitatively, we see no effect of strength of selection. With weaker selection, the proportion of cooperators is closer to 0.5, because then, drift becomes more important than selection, and there is negligible difference in the fitness of cooperators and defectors. See Fig S2E-F.

S5.4 Effect of total population size

Most of our simulations have used 1024 individuals. One may suspect that with much larger population sizes, the stochastic effects on group compositions will average out and cooperators with get no selective advantage. However, because of the spatial extent of the system also increases proportionally to the population size, such averaging out does not occur. Instead, we see that the evolved proportion of cooperators increases with population size and converges to a certain value (Fig

S2G). For these simulations, we used $c = 0.1$ and $c_s = 2$, and scaled the system size to keep the density of individuals constant. We may treat this value as the actual evolved proportion. Thus, our simulation in fact slightly underestimate the evolved proportion of cooperators, and larger scale simulations, such as with 16384 individuals, give more accurate results.

S5.5 Effect of mutation rate

Mutations play an important role in our coevolutionary mechanism. First, they provide the initial bias in ΔR_s, which cooperators capitalize upon to initiate the arms race. Second, some amount of mutation is necessary to neutralize the negative selection induced by the cost of cooperation. It is much lower in this case, however, than what would be necessary in the absence of evolvable flocking interactions.

For numerical stability, we have used a higher mutation rate than what is typically observed in nature. However, the model does not necessarily assume the mutation rate to be genetic, but can also capture random fluctuations in strategies of individuals. Under this interpretation, the mutation rate used in our simulations may not be completely unrealistic. However, such a choice of high mutation rate demands that robustness to mutation rate also be checked. We compare the evolved proportion of cooperators for different values of mutation rate with the expected number of cooperators under selection-mutation equilibrium. This expected number is calculated semi-analytically assuming absence of any cyclical or arms-race dynamics, as shown in section S3.4.

We find that the observed proportion is greater than that expected (Fig S2H), suggesting that the arms-race dynamics created by spontaneous self-sorting allows cooperators to persist in greater numbers than what only mutations allow.

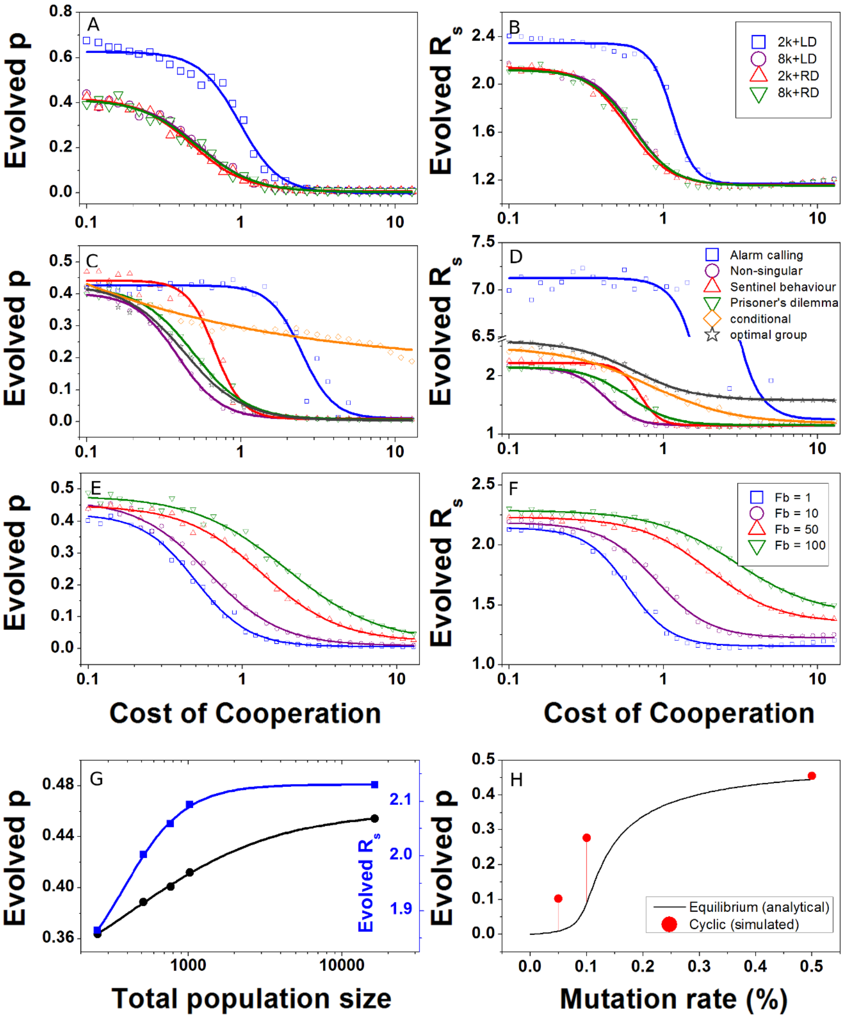

Figure S 2: Robustness of results to model variations. Parameters: $c_s = 2$. Other parameters as in Table A (see Methods).

References

[1] Guttal V, Couzin ID. Social interactions, information use, and the evolution of collective migration. Proceedings of the National Academy of Sciences. 2010;107(37):16172–16177. doi:10.1073/pnas.1006874107.

[2] Ioannou CC, Guttal V, Couzin ID. Predatory Fish Select for Coordinated Collective Motion in Virtual Prey. Science. 2012;337(6099):1212–1215. doi:10.1126/science.1218919.

[3] Torney C, Neufeld Z, Couzin ID, Levin SA. Context-Dependent Interaction Leads to Emergent Search Behavior in Social Aggregates. Proceedings of the National Academy of Sciences of the United States of America. 2009;106(52):22055–22060. doi:10.1073/pnas.0907929106.

[4] Gardiner CW. Handbook of stochastic methods. vol. 4. Springer Berlin; 1985.

[5] Cormen TH. Introduction to algorithms. MIT press; 2009.

[6] Wilson DS. A theory of group selection. Proceedings of the National Academy of Sciences. 1975;72(1):143–146.

[7] PEPPER JW. Relatedness in Trait Group Models of Social Evolution. Journal of Theoretical Biology. 2000;206(3):355 – 368. doi:http://dx.doi.org/10.1006/jtbi.2000.2132.

[8] Axelrod R, Hamilton WD. The evolution of cooperation. Science. 1981;211(4489):1390–1396. doi:10.1126/science.7466396.

[9] McElreath R, Boyd R. Mathematical models of social evolution: A guide for the perplexed. University of Chicago Press; 2008.
