# Supplementary material for: Mobility can promote the evolution of cooperation via emergent self-assortment dynamics
Source: PLoS Comput Biol. 2017 Sep 8;13(9):e1005732. doi: 10.1371/journal.pcbi.1005732 (PMC5607214; doi:10.1371/journal.pcbi.1005732)
Supplement: S6 Appendix — (PDF) [file pcbi.1005732.s006.pdf]

S6 Appendix Passive particles system

Simulations of the passive system show the same features as the active system. Fig S3 shows the features of the coevolutionary mechanism observed in the passive system, corresponding to those of Fig 3 in the main text.

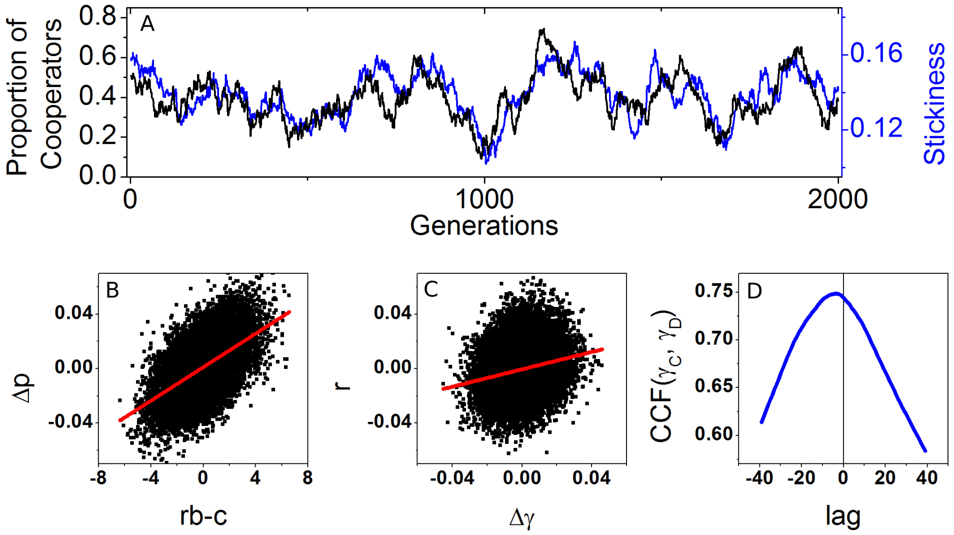

Figure S 3: Fig 3 in the main text showed the mechanism of coevolution for active case. The exact same results are obtained in the passive case, as shown in this figure. Parameters: $c = 0.1$, $b = 100$, $c_s = 100$, $\mu = 0.1$, $\lambda_0 = 1$. Other parameters as in Table A (see Methods).

We add a remark about the importance of cost of cohesion. It is evident from comparing Fig 2 of the main text that the cost of cohesion plays has a stronger quantitative effect in the active system than in the passive system (e.g. slope of the lines in Fig 2C versus Fig 2D of main text). This is because of the following reason: In the active system, individuals with large values of cohesion (R_s, the radius of interaction) form groups of large sizes in which defectors have a relative advantage. Therefore, a cost of cohesion that prevents run-away selection for large R_s is crucial to maintain cooperation. In contrast, even with large values of cohesion, individuals in the passive systems can not form large groups because the underlying turbulent dynamic leads to fission of large sized groups. Hence, the cost of cohesion that limits evolution of cohesion is less important.

References

[1] Guttal V, Couzin ID. Social interactions, information use, and the evolution of collective migration. Proceedings of the National Academy of Sciences. 2010;107(37):16172–16177. doi:10.1073/pnas.1006874107.

[2] Ioannou CC, Guttal V, Couzin ID. Predatory Fish Select for Coordinated Collective Motion in Virtual Prey. Science. 2012;337(6099):1212–1215. doi:10.1126/science.1218919.

[3] Torney C, Neufeld Z, Couzin ID, Levin SA. Context-Dependent Interaction Leads to Emergent Search Behavior in Social Aggregates. Proceedings of the National Academy of Sciences of the United States of America. 2009;106(52):22055–22060. doi:10.1073/pnas.0907929106.

[4] Gardiner CW. Handbook of stochastic methods. vol. 4. Springer Berlin; 1985.

[5] Cormen TH. Introduction to algorithms. MIT press; 2009.

[6] Wilson DS. A theory of group selection. Proceedings of the National Academy of Sciences. 1975;72(1):143–146.

[7] PEPPER JW. Relatedness in Trait Group Models of Social Evolution. Journal of Theoretical Biology. 2000;206(3):355 – 368. doi:http://dx.doi.org/10.1006/jtbi.2000.2132.

[8] Axelrod R, Hamilton WD. The evolution of cooperation. Science. 1981;211(4489):1390–1396. doi:10.1126/science.7466396.

[9] McElreath R, Boyd R. Mathematical models of social evolution: A guide for the perplexed. University of Chicago Press; 2008.
