# Supplementary material for: Mobility can promote the evolution of cooperation via emergent self-assortment dynamics
Source: PLoS Comput Biol. 2017 Sep 8;13(9):e1005732. doi: 10.1371/journal.pcbi.1005732 (PMC5607214; doi:10.1371/journal.pcbi.1005732)
Supplement: S7 Appendix — (PDF) [file pcbi.1005732.s007.pdf]

S7 Appendix Analytical calculations of the simplified binary-trait model

To further analyse our coevolutionary mechanism and to develop a general theory underlying our results, we develop an analytical version of our simplified model with binary traits. This section is devoted to the description and analysis of this analytical model.

Consider a population of N individuals. Each individual i has two binary traits, viz. cooperative tendency $\omega_{c,i}$ and cohesive tendency $\omega_{s,i}$. Cooperators have $\omega_{c,i} = 1$ and defectors have $\omega_{c,i} = 0$. Individuals with $\omega_{s,i} = 1$ have a substantial cohesive tendency and are likely to form groups with other cohesive individuals, and those with $\omega_{s,i} = 0$ have no cohesive tendency and are likely to remain solitary. In this section, for notational ease we will also refer to the cohesive tendency as 'flocking tendency'.

Since both traits are binary, there are four possible types of individuals in the population corresponding to the four possible (ω_c, ω_s) pairs: solitary defectors (0,0), flocking defectors (0,1), solitary cooperators (1,0), and flocking cooperators (1, 1). Let the numbers of these types respectively, be $N_{sd}, N_{fd}, N_{sc}, N_{fc}$ where subscript f stands for flocking, s for solitary, c for cooperator, and d for defector. Also, the number of flocking and solitary individuals is then $N_f = N_{fc} + N_{fd}$ and $N_s = N_{sc} + N_{fd}$. Note that $N_{sd} + N_{fd} + N_{sc} + N_{fc} = N$. We hold the population size constant, and thus the frequencies of any 3 types automatically determine frequency of the fourth type.

Let the proportion of flocking individuals in the population be q. Thus, there are $N_f = qN$ flocking individuals and $N_s = (1-q)N$ solitary individuals. Let the proportion of cooperators in any category z be p_z. Thus, the proportion of cooperators among solitary individuals is p_s $(= N_{sc}/N_s)$ and that among flocking individuals is p_f $(= N_{fc}/N_f)$. The total proportion of cooperators in the population (p) is

$$p = \frac{p_f qN + p_s(1-q)N}{qN + (1-q)N} = p_f q + p_s(1-q) \tag{S7.1}$$

We treat p_f, p_s and q as the state variables of our system. The numbers of all the four types in the population can be expressed in terms of these three variables as follows:

$$\begin{aligned} N_{sc} &= p_s(1-q)N \\ N_{sd} &= (1-p_s)(1-q)N \\ N_{fc} &= p_f q N \\ N_{fd} &= (1-p_f)qN \end{aligned} \tag{S7.2}$$

Now, we recall the general replicator equation for two traits. If the population has two traits 1 and 2, then the change in frequency of trait 1 (p_1) is

$$V\frac{dp_1}{dt} = p_1(1-p_1)(V_1 - V_2) \tag{S7.3}$$

where V is the average fitness of the population, and V_i is the average fitness of individuals with trait i. We apply this equation to write the time evolution of our three state variables:

$$\begin{aligned} V_f\frac{dp_f}{dt} &= p_f(1-p_f)(V_{fc} - V_{fd}) \\ V_s\frac{dp_s}{dt} &= p_s(1-p_s)(V_{sc} - V_{sd}) \\ V\frac{dq}{dt} &= q(1-q)(V_f - V_s) \end{aligned} \tag{S7.4}$$

where V_z is the average fitness of the appropriate type indicated by subscripts. Absence of subscript indicates a quantity for the whole population. We have summarized all the key symbols in Table C.

The first two equations in the above system capture dynamics of cooperators within solitary and flocking sub-populations independently of each other, by capturing the change in proportion of cooperators only within the sub-population. The third equation captures the relative dynamics of the two sub-populations.

We calculate the payoff differences mentioned above. For ease of calculations, we use the non-singular payoffs (Eq S5.5) for this section. The payoff of any individual i in a group of size n_g with k_g cooperators is

$$V_i = V_0 + \frac{k_g - \omega_{c,i}}{n_g}b - c\omega_{c,i} - c_s\omega_{s,i} \tag{S7.5}$$

where we have added the extra cost of flocking c_s for flocking individuals.

Thus, payoffs of solitary individuals are

$$
\begin{aligned}
V_{sc} &= V_0 - c \\
V_{sd} &= V_0 \\
V_s &= p_s(V_0 - c) + (1 - p_s)V_0 = V_0 - cp_s
\end{aligned}
\tag{S7.6}
$$

We now calculate the payoffs for flocking individuals. In our spatial model, cohesive individuals form groups of varying sizes, with mean group size depending on the magnitude of the cohesive tendency. In this discrete version of the model, cohesive individuals all have a fixed cohesive (flocking) tendency. As a result, we assume that they form groups (flocks) which follow a distribution with mean group size n.

Then, from equation S4.12 in section S4 Appendix,

$$
V_{fc} - V_{fd} = r_{\text{groups}} b - c \tag{S7.7}
$$

However, since all flocking individuals have the same flocking tendency, the average assortment among groups (r_{groups}) is zero. We shall use r_{groups} to be a Gaussian random variable with mean 0 and standard-deviation σ_{rf}. The assortment that we are interested in is the one arising from differential flocking tendencies of cooperators and defectors, which, in this discrete model, manifest as differential proportions of flocking cooperators and defectors (as we shall show shortly).

Now,

$$
V_f = E[V_g] = V_0 + p_f(b - c) - bE[\frac{p_g}{n_g}] - c_s \tag{S7.8}
$$

To make our equations analytically tractable, we may make a further simplification: we assume that all cohesive individuals form groups of fixed size n. Then the above equation reduces to

$$
\begin{aligned}
V_f &= V_0 + p_f(b - c) - b\frac{p_f}{n} - c_s \\
&= V_0 + p_f(b\nu - c) - c_s
\end{aligned}
\tag{S7.9}
$$

where $\nu = (n - 1)/n$.

Finally, the average fitness of the entire population V can be calculated as

$$
\begin{aligned}
V &= \frac{V_f N_f + V_s N_s}{N} \\
&= V_f q + V_s(1 - q)
\end{aligned}
\tag{S7.10}
$$

Plugging equations S7.9, S7.6 and S7.10 into our replicator equation S7.4, we get

$$\begin{aligned}\frac{dp_f}{dt} &= p_f(1-p_f)\frac{r_{\text{groups}}b - c}{V_0 + p_f(b\nu - c) - c_s}\\ \frac{dp_s}{dt} &= -\,p_s(1-p_s)\frac{c}{V_0 - p_s c}\\ \frac{dq}{dt} &= q(1-q)\frac{p_f(b\nu - c) + cp_s - c_s}{V_0 + q(p_f(b\nu - c) - c_s) - (1-q)cp_s}\end{aligned} \tag{S7.11}$$

Equations S7.11 can be further simplified if we take the weak selection limit. Under this assumption, the additional terms in the denominator are much smaller than V_0, and can be neglected. The system then becomes

$$\begin{aligned}\frac{dp_f}{dt} &\approx p_f(1-p_f)(r_{\text{groups}}b - c)\\ \frac{dp_s}{dt} &\approx \ -\,p_s(1-p_s)c\\ \frac{dq}{dt} &\approx q(1-q)(p_f(b\nu - c) + cp_s - c_s)\end{aligned} \tag{S7.12}$$

where V_0 has been absorbed in the other parameters b, c, and c_s.

We now analytically calculate the differential cohesive tendency $\Delta\omega_s$, which indeed, is at the heart of assortment. In this discrete version, $\Delta\omega_s$ manifests in terms of the frequencies of cohesive and solitary cooperators and defectors, as follows:

$$\begin{aligned}\Delta\omega_s &= \frac{p_f q \times 1 + p_s(1-q)\times 0}{p_f q + p_s(1-q)} - \frac{(1-p_f)q \times 1 + (1-p_s)(1-q)\times 0}{(1-p_f)q + (1-p_s)(1-q)}\\ &= q(1-q)\frac{p_f - p_s}{(qp_f + (1-q)p_s)(1 - qp_f - (1-q)p_s)}\\ &= \frac{q(1-q)}{p(1-p)}(p_f - p_s)\end{aligned} \tag{S7.13}$$

Finally, we write the equation for change in the total proportion of cooperators in the population p, which is the main variable of interest:

$$
\begin{aligned}
\frac{dp}{dt} &= q\frac{dp_f}{dt} + p_f\frac{dq}{dt} + (1-q)\frac{dp_s}{dt} - p_s\frac{dq}{dt} \\
&= q\frac{dp_f}{dt} + (p_f - p_s)\frac{dq}{dt} + (1-q)\frac{dp_s}{dt} \\
&= -cqp_f(1-p_f) - c(1-q)p_s(1-p_s) \\
&\quad + (p_f - p_s)q(1-q)(p_f(b\nu - c) + cp_s - c_s) \\
&= -cqp_f(1-p_f) - c(1-q)p_s(1-p_s) \\
&\quad + p(1-p)\Delta\omega_s(p_f(b\nu - c) + cp_s - c_s)
\end{aligned} \tag{S7.14}
$$

We can now define assortment. The above equation can be re-written in the form of a replicator equation for p

$$
\frac{dp}{dt} = p(1-p)(rb_{\text{eff}} - c_{\text{eff}}) \tag{S7.15}
$$

Where r is assortment, b_{eff} is the effective net benefit of cooperation and cohesion, and c_{eff} is the effective cost of cooperation, as follows:

$$
\begin{aligned}
r &= \Delta\omega_s \\
c_{\text{eff}} &= -c\Big(\frac{qp_f(1-p_f) + (1-q)p_s(1-p_s)}{p(1-p)}\Big) \\
b_{\text{eff}} &= p_f(b\nu - c) + cp_s - c_s
\end{aligned} \tag{S7.16}
$$

This equation easily shows how differential cohesion leads to assortment.

In the main text, we have shown results of numerically evolving equations S7.12 with mutations, as follows:

$$
\begin{aligned}
\frac{dp_f}{dt} &= -p_f(1-p_f)c + \sigma_r\eta_{11} + \sigma_u\eta_{12} \\
\frac{dp_s}{dt} &= -p_s(1-p_s)c + \sigma_s\eta_2 \\
\frac{dq}{dt} &= q(1-q)(p_f(b\nu - c) + cp_s - c_s) + \sigma_q\eta_3
\end{aligned} \tag{S7.17}
$$

where σs are magnitudes of noise due to mutation and ηs are standard Gaussian random variables. r_{groups} is chance assortment occurring due to fluctuations in

group compositions, and is modelled as a Gaussian random variable with mean zero and variance σ_{rf}^2. The σ terms are assumed to have forms as in Equation S7.18. σ_r is the stochastic term that absorbs the r_{groups} term, calculated by some algebraic reorganization of equation S7.12.

$$\begin{aligned}
\sigma_r &= p_f(1-p_f)\sigma_{\mathrm{rf}} b \\
\sigma_u &= \frac{\mu_f}{q} \\
\sigma_s &= \frac{\mu_s}{1-q} \\
\sigma_q &= \mu_q
\end{aligned} \tag{S7.18}$$

where μ's are mutation rates. We divide the mutation term by the proportion of cohesive / solitary individuals to account for the fact that when the proportion of the cohesive (or solitary) type is very low, even a single mutation may cause a large change in the p_f (or p_s). This reasonably describes dynamics for finite sized populations, or of infinite populations extended in space, such that the interactions extend only over a finite distance. We use a gaussian noise to model mutations to account for fluctuations in p_f and p_s due to mutations, or due to cooperation arising from chance encounters of genetically solitary individuals. These features are indeed observed in our explicitly spatial version of this discrete-trait model (Figs S5 and S6).

The parameters used for Figure 4 in the main text are listed in the 'SDE' column in Table C. Note: In the equation shown in the main text, we have combined the stochastic terms σ_r and σ_u into a single term, as: $\sigma_r\eta_{11} + \sigma_u\eta_{12} = \sigma_f\eta_1$, where $\sigma_f = \sqrt{\sigma_r^2 + \sigma_u^2}$, but retained them separately in numerical integration.

It is possible to analytically solve the above system for fixed points and find their stability, if we assume that the mutation term is of the form $\mu(1-2z)$ for variable z, so that mutations cause any trait to increase when rare and decrease when abundant. This deterministic approximation which has no noise terms is unrealistic for small populations, but reasonable for large populations and useful to gain insight in the dynamics of the system. The system then becomes an ordinary differential equation as follows:

$$
\begin{aligned}
\frac{dp_f}{dt} &= -\, p_f(1-p_f)c + \frac{\mu_f(1-2p_f)}{q} \\
\frac{dp_s}{dt} &= -\, p_s(1-p_s)c + \frac{\mu_s(1-2p_f)}{1-q} \\
\frac{dq}{dt} &= q(1-q)(p_f(b\nu - c) + cp_s - c_s) + \mu_q(1-2q)
\end{aligned}
\tag{S7.19}
$$

The behaviour of this system is shown in Fig S4. For the parameter values as in the 'ODE' column in Table C, It has a single fixed point, and the eigenvalues have a finite imaginary part and a negative real part, so that the system spirals to this fixed point. For the run shown, the fixed point is $(0.34, 0.14, 0.2)$ and the eigenvalues are $-0.02 \pm 0.35i$, and -0.08.

**Parameter/constant**	**Symbol**	**Val (ODE)**	**Val (SDE)**
**Dynamical variables**			
Proportion of cooperators among flocks	p_f	-	-
Proportion of cooperators among solitary	p_s	-	-
Proportion of flocking individuals	q	-	-
**Subscripts**			
Entire population	none	-	-
Flocking (cohesive) individuals	$\square_f$	-	-
Solitary (non-cohesive) individuals	$\square_s$	-	-
Flocking cooperators	$\square_{fc}$	-	-
Solitary cooperators	$\square_{sc}$	-	-
Flocking defectors	$\square_{fd}$	-	-
Solitary defectors	$\square_{sd}$	-	-
**Individual traits**			
Cooperative tendency	$\omega_{c,i}$	-	-
Cohesive tendency	$\omega_{s,i}$	-	-
Fitness	V	-	-
**Parameters**			
Benefit from cooperators	b	100	100
Cost of cooperation	c	0.1	0.1
Cost of cohesion	c_s	~ 27	~ 50
Mutation rate in p_f	μ_f	10^{-3}	2×10^{-6}
Mutation rate in p_s	μ_s	10^{-3}	2×10^{-6}
Mutation rate in q	μ_q	10^{-5}	10^{-7}
Group size of flocking individuals	n	5	5
Time step for numerical integration	-	0.005	0.005
Standard deviation of r_{groups}	σ_{rf}	-	0.002

Table C: Symbols and parameter values for the analytical model.

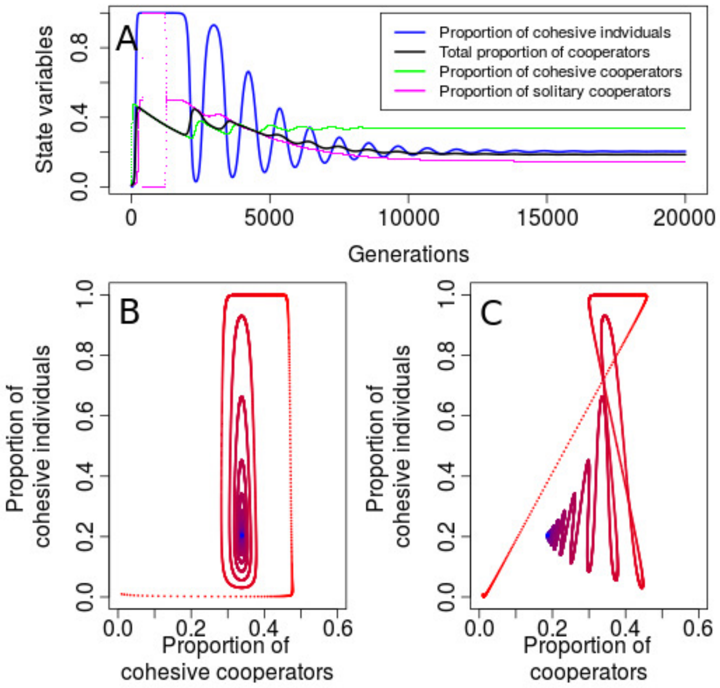

Figure S 4: The time evolution (A) and phase portraits (B-C) of equations S7.19. In A, q in blue, p_f in green, p_s in magenta, and p in black. In B-C, generations increase from red to blue. The phase portrait C resembles that in the main text.

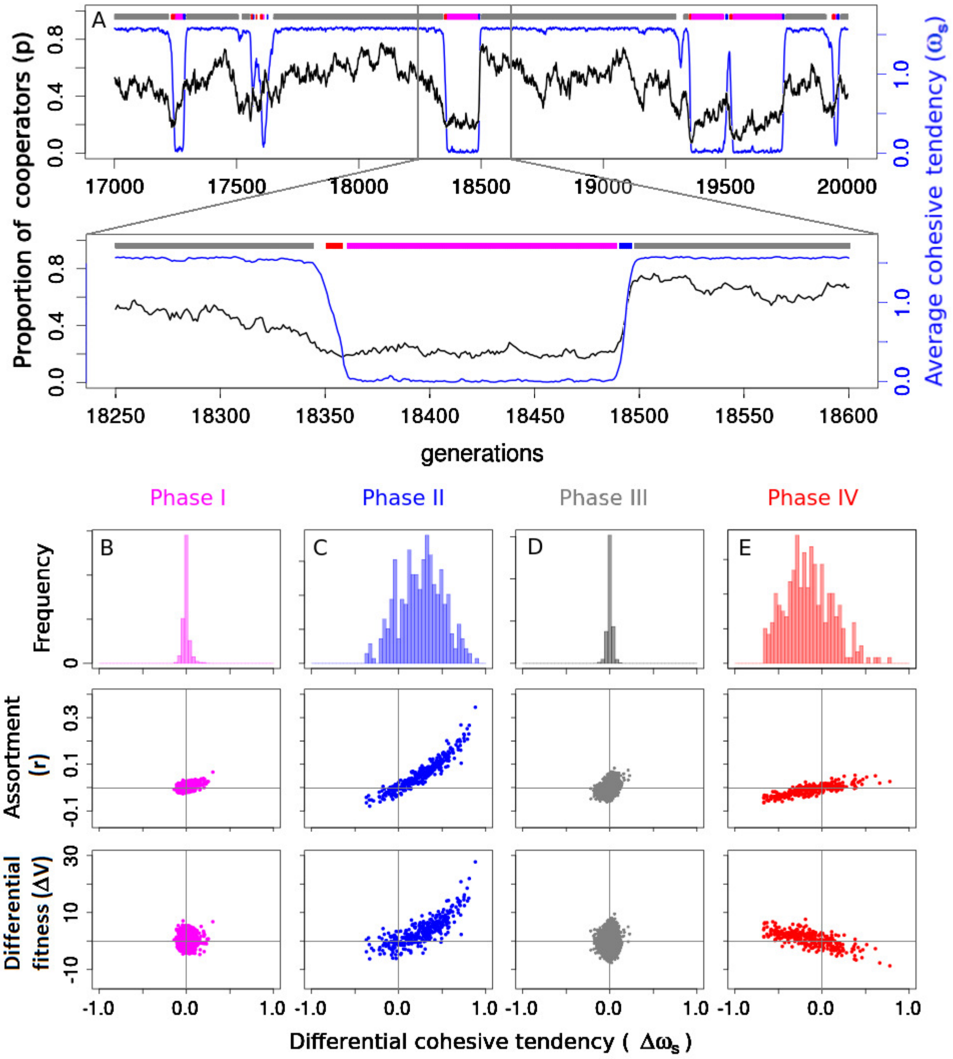

Figure S 5: Results of explicit spatial simulation of the model with binary cohesive trait (using the active system). Notice how the results match with the analytical model shown in the main text. (A) Four phases of cyclical arms race for costly cohesion (ω_s; blue) and cooperation (p; black). The four phases are evident from both full and zoomed in time series of ω_s marked by the coloured bands along the generation axis. (B) Phase I (magenta): ω_s is close to zero with no differential cohesion ($\Delta\omega_s = \omega_{sc} - \omega_{sd} \approx 0$), thus no assortment ($r \approx 0$) and no differential fitness ($\Delta V = V_c - V_d \approx 0$) benefit to cooperators. (C) Phase II (blue): Cooperators lead an arms race to have a higher (costly) ω_s since they benefit by differential cohesion and assorting; but defectors benefit by matching ω_s of cooperators. (D) Phase III (grey): ω_s is high and same for all individuals, thus no assortment and no differential fitness benefit to cooperators. (E) Phase IV (red): Cooperators and defectors reduce their cohesive tendencies to avoid costs of cohesion (decreasing ω_s) and returning to Phase I. The cyclical dynamics continues, as seen in (A). Parameters: $c = 0.1$, $b = 100$, $c_s \approx 25$. For flocking individuals, $R_s = 2.6$. Other parameters as in Table A.

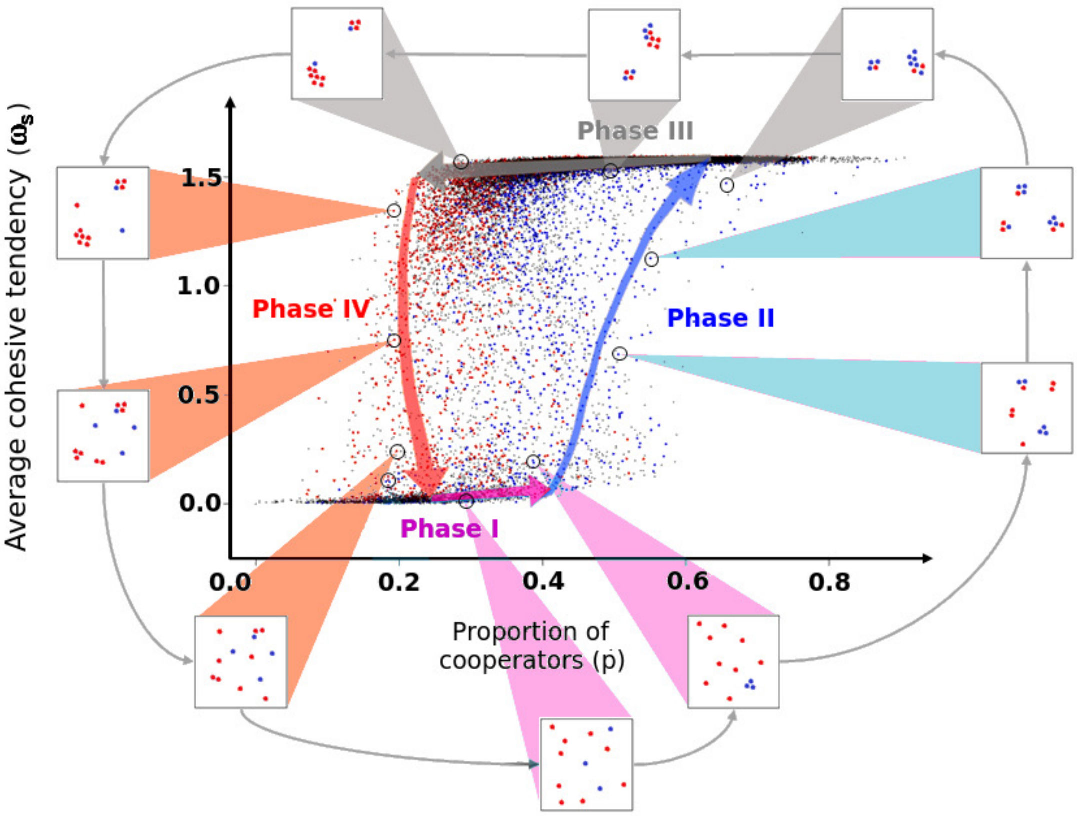

Figure S 6: Cyclical coevolutionary dynamics of costly cooperative and cohesive traits along with schematic snapshots of spatial dynamics. Each data point (p,ω_s) represents the instantaneous values of the proportion of cooperators (p) and average cohesive tendency (ω_s) from the time series shown in Fig S5A. The four phases are evident here too and are coloured as in Fig S5. We show snapshots representative of spatial structure of the population (exaggerated for visual guidance), with cooperators in blue and defectors in red. In phase I, all individuals have low (and no) cohesive tendencies, leading to a well mixed system of 'random walkers'. Accidental group formation of cohesive and cooperative individuals initiates the arms race to increase costly ω_s, leading to Phase II which exhibits spatial sorting driven by differential cohesion. Nearly all individuals are cohesive in Phase III, destroying assortment and thus, reducing the level of cooperation. Finally, cooperators and defectors reduce the costly cohesive tendencies, thus reaching the Phase I back where p and ω_s are both low. These four phases repeat over generations. Thus, costly cohesive and emergent self-sorting group dynamics maintain an average intermediate values of p and ω_s which are higher than expected by various baseline scenarios (shown in Fig 1).

References

[1] Guttal V, Couzin ID. Social interactions, information use, and the evolution of collective migration. Proceedings of the National Academy of Sciences. 2010;107(37):16172–16177. doi:10.1073/pnas.1006874107.

[2] Ioannou CC, Guttal V, Couzin ID. Predatory Fish Select for Coordinated Collective Motion in Virtual Prey. Science. 2012;337(6099):1212–1215. doi:10.1126/science.1218919.

[3] Torney C, Neufeld Z, Couzin ID, Levin SA. Context-Dependent Interaction Leads to Emergent Search Behavior in Social Aggregates. Proceedings of the National Academy of Sciences of the United States of America. 2009;106(52):22055–22060. doi:10.1073/pnas.0907929106.

[4] Gardiner CW. Handbook of stochastic methods. vol. 4. Springer Berlin; 1985.

[5] Cormen TH. Introduction to algorithms. MIT press; 2009.

[6] Wilson DS. A theory of group selection. Proceedings of the National Academy of Sciences. 1975;72(1):143–146.

[7] PEPPER JW. Relatedness in Trait Group Models of Social Evolution. Journal of Theoretical Biology. 2000;206(3):355 – 368. doi:http://dx.doi.org/10.1006/jtbi.2000.2132.

[8] Axelrod R, Hamilton WD. The evolution of cooperation. Science. 1981;211(4489):1390–1396. doi:10.1126/science.7466396.

[9] McElreath R, Boyd R. Mathematical models of social evolution: A guide for the perplexed. University of Chicago Press; 2008.
