# Supplementary figures and images for: Mobility can promote the evolution of cooperation via emergent self-assortment dynamics
Source: PLoS Comput Biol. 2017 Sep 8;13(9):e1005732. doi: 10.1371/journal.pcbi.1005732 (PMC5607214; doi:10.1371/journal.pcbi.1005732)

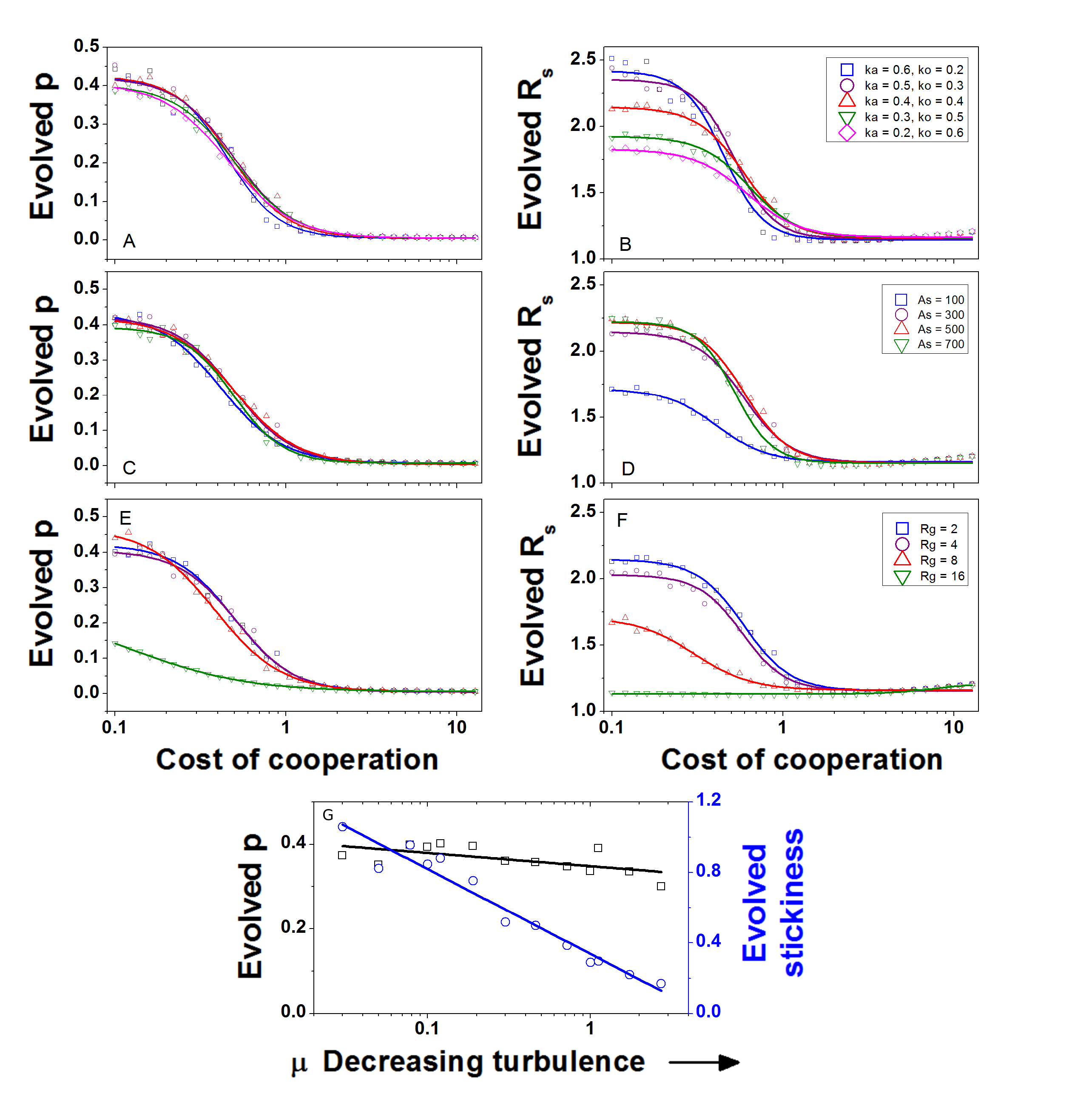

Supplement: S1 Fig — (PNG) [file pcbi.1005732.s010.png]

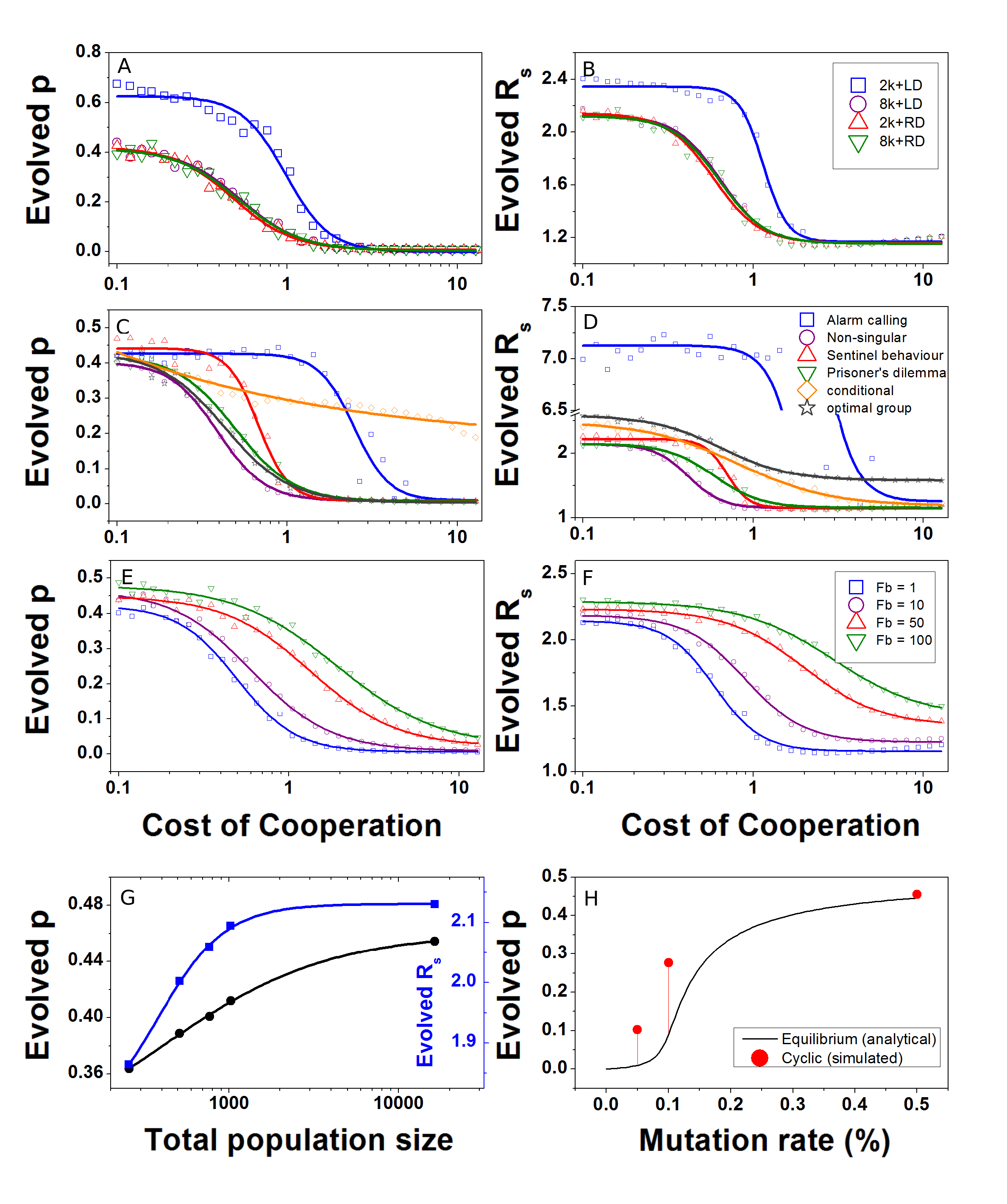

Supplement: S2 Fig — (PNG) [file pcbi.1005732.s011.png]

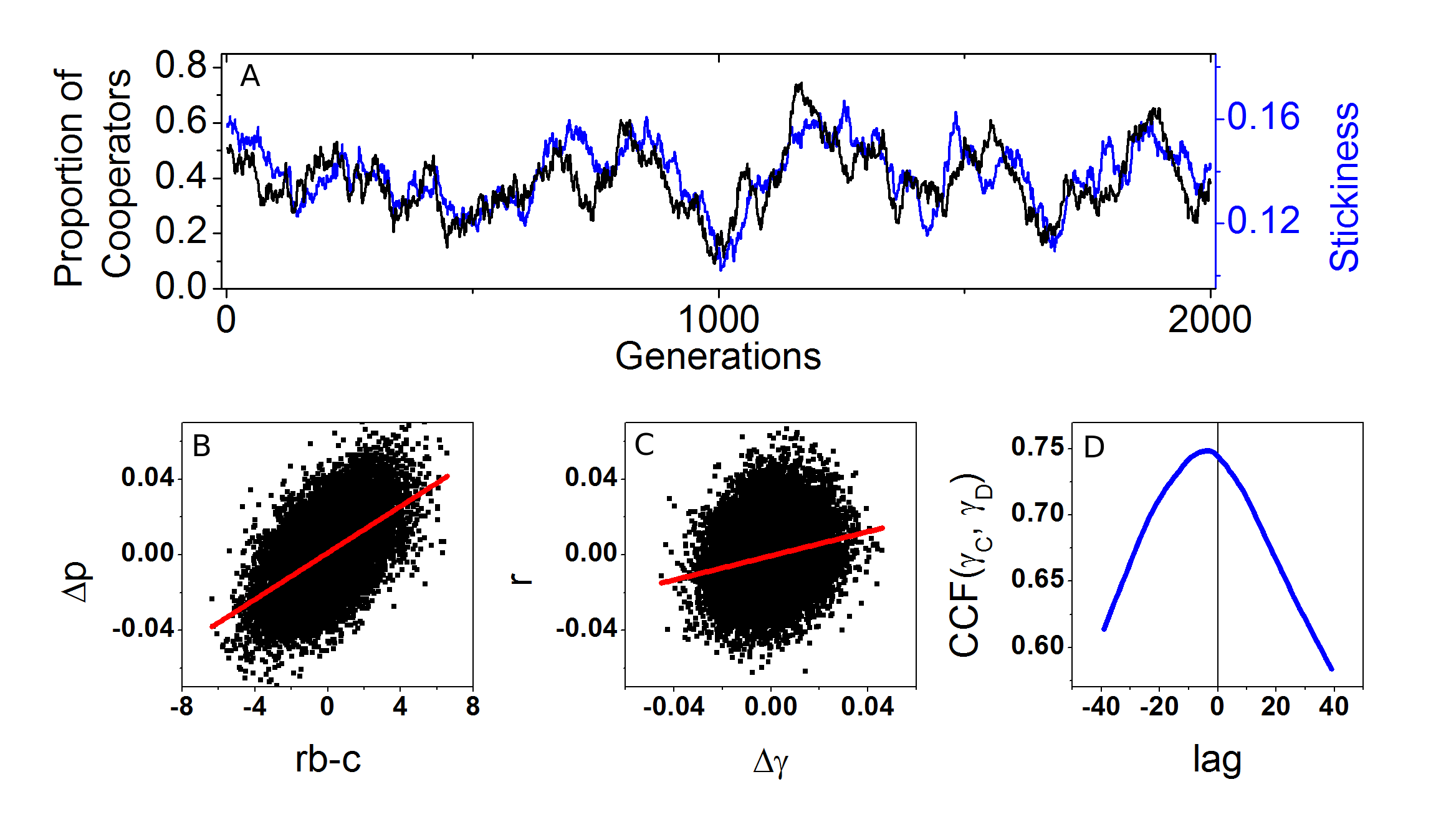

Supplement: S3 Fig — (PNG) [file pcbi.1005732.s012.png]

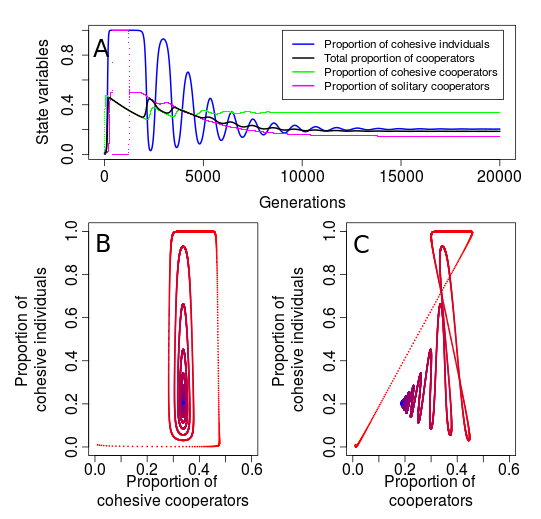

Supplement: S4 Fig — (PNG) [file pcbi.1005732.s013.png]

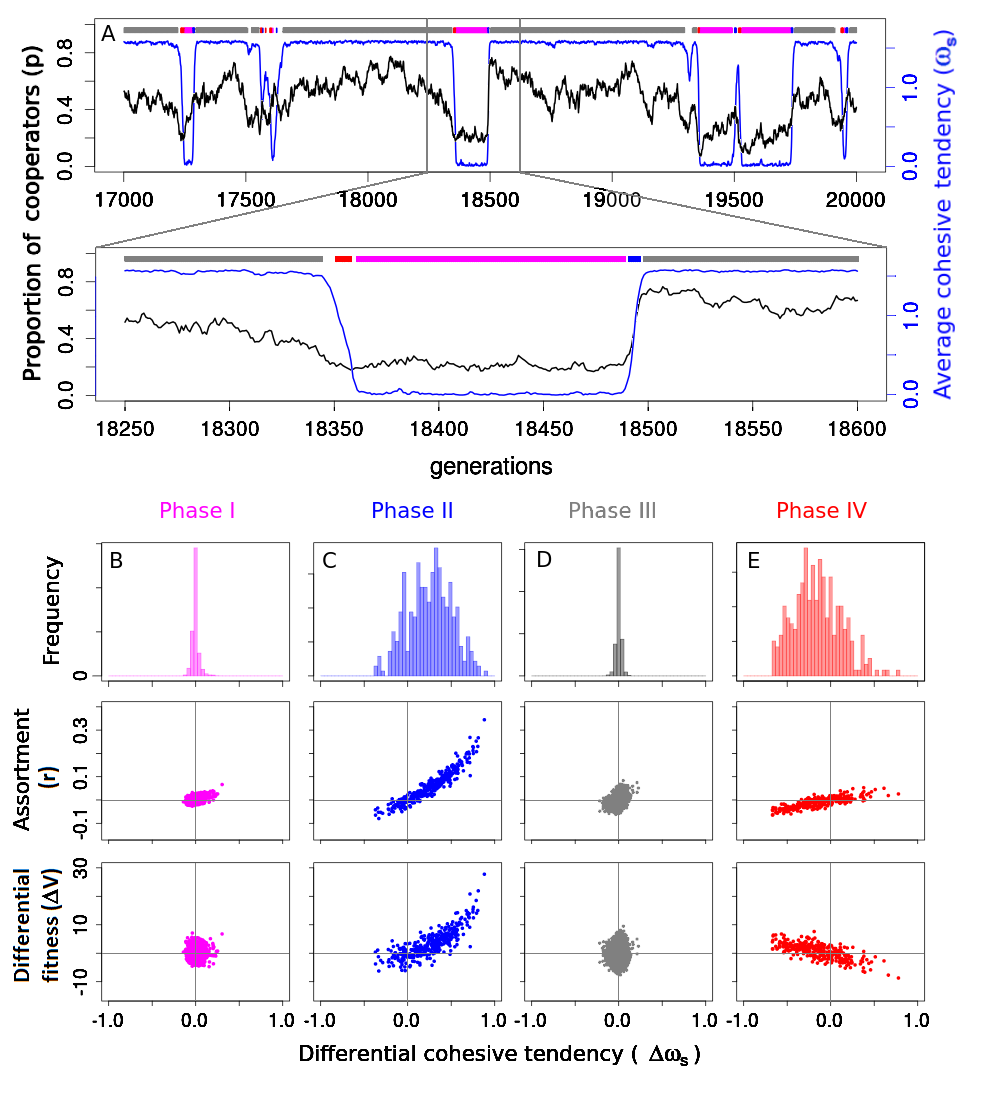

Supplement: S5 Fig — (PNG) [file pcbi.1005732.s014.png]

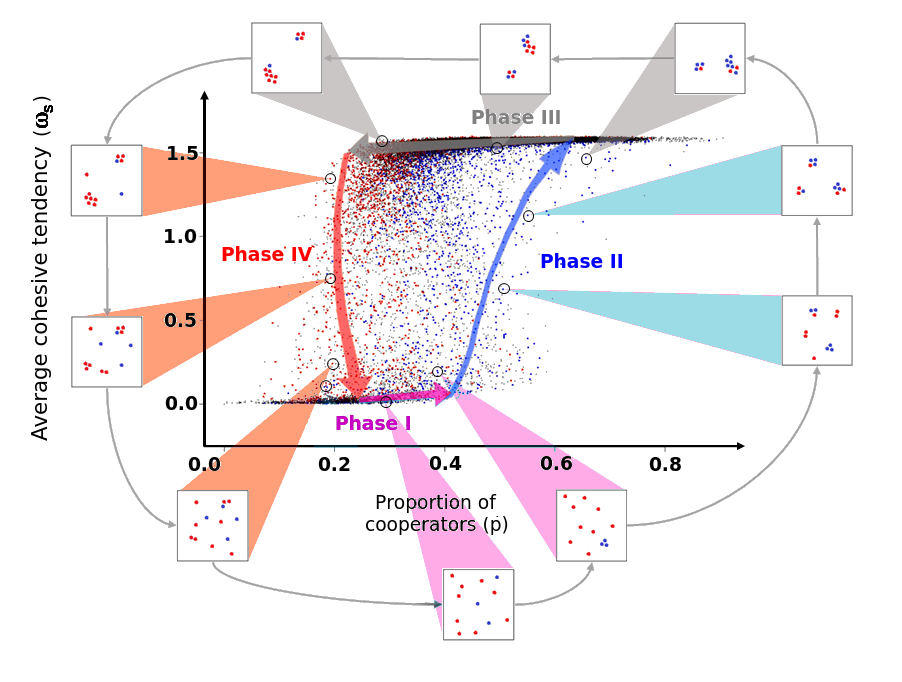

Supplement: S6 Fig — (PNG) [file pcbi.1005732.s015.png]
